# Retinoic acid-induced 1 gene haploinsufficiency alters lipid metabolism and causes autophagy defects in Smith-Magenis syndrome

**DOI:** 10.1038/s41419-022-05410-7

**Published:** 2022-11-21

**Authors:** Elisa Maria Turco, Angela Maria Giada Giovenale, Laura Sireno, Martina Mazzoni, Alessandra Cammareri, Caterina Marchioretti, Laura Goracci, Alessandra Di Veroli, Elena Marchesan, Daniel D’Andrea, Antonella Falconieri, Barbara Torres, Laura Bernardini, Maria Chiara Magnifico, Alessio Paone, Serena Rinaldo, Matteo Della Monica, Stefano D’Arrigo, Diana Postorivo, Anna Maria Nardone, Giuseppe Zampino, Roberta Onesimo, Chiara Leoni, Federico Caicci, Domenico Raimondo, Elena Binda, Laura Trobiani, Antonella De Jaco, Ada Maria Tata, Daniela Ferrari, Francesca Cutruzzolà, Gianluigi Mazzoccoli, Elena Ziviani, Maria Pennuto, Angelo Luigi Vescovi, Jessica Rosati

**Affiliations:** 1 Cellular Reprogramming Unit, Fondazione IRCCS Casa Sollievo della Sofferenza, Viale dei Cappuccini, 71013 San Giovanni Rotondo, FG Italy; 2 Department of Biotechnology and Biosciences, University of Milano-Bicocca, Piazza della Scienza 2, 20126 Milano, Italy; 3 Veneto Institute of Molecular Medicine (VIMM), via Orus 2, 35129 Padova, Italy; 4 Department of Biomedical Sciences, University of Padova, via Ugo Bassi 58/B, 35131 Padova, Italy; 5 Department of Chemistry, Biology, and Biotechnology, University of Perugia, Via Elce di Sotto 8, 06123 Perugia, Italy; 6 Department of Biology, University of Padova, Via U. Bassi 58/b, 35121 Padova, Italy; 7 Interdisciplinary Biomedical Research Centre, School of Science and Technology, Nottingham Trent University, Clifton, NG11 8NS UK; 8 Medical Genetics Unit, Fondazione IRCCS Casa Sollievo della Sofferenza, Viale dei Cappuccini, 71013 San Giovanni Rotondo, Italy; 9 Department of Biochemical Sciences, “A. Rossi Fanelli”, University of Rome “La Sapienza”, P.le Aldo Moro 5, 00185 Rome, Italy; 10 UOC Genetica Medica e di Laboratorio, AORN “A. Cardarelli”, Via Antonio Cardarelli 9, 80131 Napoli, Italy; 11 Department of Pediatric Neuroscience, Fondazione IRCCS Istituto Neurologico Carlo Besta, Via Giovanni Celoria, 11, 20133 Milano, Italy; 12 Medical Genetics Laboratory, “Policlinico Tor Vergata” Hospital, Viale Oxford 81, 00133 Rome, Italy; 13 Rare Diseases and Birth Defects Unit, Fondazione Policlinico Universitario Agostino Gemelli IRCCS, Largo Agostino Gemelli 8, 00168 Rome, Italy; 14 Dipartimento di Scienze della Vita e Sanità Pubblica, Università Cattolica del S. Cuore, Largo Francesco Vito, 1, 00168 Rome, Italy; 15 Department of Biology, DiBio Imaging Facility, University of Padova, Via U. Bassi 58/b, 35121 Padova, Italy; 16 Department of Molecular Medicine, University of Rome “La Sapienza”, Viale Regina Elena 324, 00161 Rome, Italy; 17 Unit of Cancer and Stem Cells, Fondazione IRCCS Casa Sollievo della Sofferenza, Viale dei Cappuccini, 71013 San Giovanni Rotondo, FG Italy; 18 Department of Biology and Biotechnology “Charles Darwin”, University of Rome “La Sapienza”, P.le Aldo Moro 5, 00185 Rome, Italy; 19 Research Center of Neurobiology “Daniel Bovet”, University of Rome “La Sapienza”, P.le Aldo Moro 5, 00185 Rome, Italy; 20 Department of Medical Sciences, Division of Internal Medicine and Chronobiology Laboratory, Fondazione IRCCS Casa Sollievo della Sofferenza, Viale dei Cappuccini, 71013 San Giovanni Rotondo, Italy

**Keywords:** Mechanisms of disease, Disease model

## Abstract

Smith-Magenis syndrome (SMS) is a neurodevelopmental disorder characterized by cognitive and behavioral symptoms, obesity, and sleep disturbance, and no therapy has been developed to alleviate its symptoms or delay disease onset. SMS occurs due to haploinsufficiency of the retinoic acid-induced-1 (*RAI1*) gene caused by either chromosomal deletion (SMS-del) or *RAI1* missense/nonsense mutation. The molecular mechanisms underlying SMS are unknown. Here, we generated and characterized primary cells derived from four SMS patients (two with SMS-del and two carrying *RAI1* point mutations) and four control subjects to investigate the pathogenetic processes underlying SMS. By combining transcriptomic and lipidomic analyses, we found altered expression of lipid and lysosomal genes, deregulation of lipid metabolism, accumulation of lipid droplets, and blocked autophagic flux. We also found that SMS cells exhibited increased cell death associated with the mitochondrial pathology and the production of reactive oxygen species. Treatment with N-acetylcysteine reduced cell death and lipid accumulation, which suggests a causative link between metabolic dyshomeostasis and cell viability. Our results highlight the pathological processes in human SMS cells involving lipid metabolism, autophagy defects and mitochondrial dysfunction and suggest new potential therapeutic targets for patient treatment.

## Introduction

Smith-Magenis syndrome (SMS, OMIM#182290) is a severe and untreatable neurodevelopmental disease with a prevalence of 1/15,000 newborns [1]. SMS is a multisystem disease characterized by behavioral, neurological, and physical abnormalities [2]. The behavioral symptoms include self-inflicted injury and stereotyped movements [3]. Most SMS patients exhibit neurological symptoms, mild-to-moderate intellectual disability, and strong long-term memory deficits [4], and almost all (96%) SMS patients also show delayed speech and motor skills [4], hyperphagia [5], and sleep disturbance [6]. Sleep disturbance is one of the earliest diagnostic indicators of SMS and manifests by frequent awakenings during night-time sleep and excessive daytime sleepiness [7]. Patients also exhibit craniofacial abnormalities [8]. A key aspect of SMS is represented by metabolic alterations and obesity that manifest before adolescence [8]. These clinical symptoms often precede compulsive eating associated with energy imbalance and dyshomeostasis of peripheral tissues. The pathological processes underlying the neurological and peripheral symptoms of SMS patients remain to be clarified.

In the majority of SMS patients (approximately 90%), the disease is linked to copy number variations (CNVs) spanning multiple genes within the 17p11.2 chromosome region [1]. Some patients with phenotypes similar to those of patients with SMS deletions (SMS-del) and carrying missense and nonsense point mutations of a single gene present in the SMS CNV, namely, the retinoic acid-induced 1 (*RAI1)* locus, have been identified, indicating a link between SMS and *RAI1* haploinsufficiency [1, 9]. Although *RAI1* haploinsufficiency is responsible for SMS, *RAI1* duplication causes a different neurodevelopmental disorder with a similar clinical presentation, namely, Potocki-Lupski syndrome (PTLS, OMIM# 610883) [10]. *RAI1* has also been associated with schizophrenia, autism and spinocerebellar ataxia type 2 [10–12]. The overlapping genetic and clinical presentations of SMS and PTLS patients clearly imply that *RAI1* is a dosage-sensitive gene whose loss of function (haploinsufficiency) or gain of function (duplication) can cause the phenotypical and neurodevelopmental alterations observed in patients. In P19 embryonal cancer cells, the *RAI1* gene was identified as one of the most highly upregulated genes during the process of neuronal differentiation induced by retinoic acid [13]. RAI1 has two nuclear localization signals (NLSs) within the carboxy-terminal domain (CTD) and localizes to both the nucleus and cytoplasm. The function of RAI1 is unknown. RAI1 associates with nucleosomes in HeLa cells [14] and regulates the expression of several genes, including circadian locomotor output cycles kaput (*CLOCK*) and brain-derived neurotrophic factor (*BDNF*) [6, 15]. However, the mechanism through which *RAI1* haploinsufficiency causes SMS is unclear.

We developed primary fibroblast cell lines derived from two SMS patients carrying different *RAI1* point mutations, two SMS-del patients, three healthy controls, and the unaffected sibling of an SMS patient. Through combined transcriptomic and lipidomic analyses, we found that loss of *RAI1* function alters lysosome and lipid gene expression and results in lipid droplet (LD) accumulation, morphological abnormalities, and enhanced cell death.

## Materials and methods

### Skin biopsy, fibroblast isolation and growth

Skin fragments from four SMS patients, a healthy sibling, and three nonparental healthy donors were cut into small fragments, plated in a drop of fetal bovine serum (FBS) on a tissue culture dish and incubated overnight at 37 °C in a 5% CO_2_ atmosphere. The fragments were maintained in Dulbecco’s modified Eagle’s medium (DMEM) with high glucose supplemented with 20% FBS, 2 mM L-glutamine, 100 U/ml penicillin‒streptomycin and 1x non-essential amino acids (Sigma Aldrich) for 20–30 days to allow fibroblast spread. Fibroblasts were isolated by trypsin dispersion, and cells were grown in the same medium and conditions. The cells were amplified and regularly cryopreserved to a maximum passage of XIII. To exclude any possible contamination by mycoplasma, the cells were regularly tested with an N-GARDE Mycoplasma PCR Reagent set (Euroclone).

### Fibroblast treatments

Fibroblasts were grown in fresh medium containing 50 μM oleic acid (OA) dissolved in chloroform for 48 h to induce LD formation. Untreated cells were incubated with an OA vehicle (chloroform) at the same concentration. Fibroblasts were grown for 24 h in medium containing 40 μM chloroquine (CQ) or 10 μM N-acetylcysteine (NAC), both dissolved in H_2_O, to inhibit autophagic flux and regulate lipid metabolism, respectively. Untreated cells were incubated in fresh culture medium.

### Immunofluorescence

Fibroblasts were plated in Cultrex® Basement Membrane Matrix (Trevigen)-pretreated coverglasses. The cells were fixed in  4% paraformaldehyde/PBS for 10 min, washed for 5 min with 1X PBS with Ca^2+^ and Mg^2+^, incubated in 100 mmol/L glycine for 10 min, permeabilized with 0.5% Triton X-100 for 5 min, and blocked with 1% bovine serum albumin for 30 min. The cells were then incubated with the following primary and secondary antibodies for 1 h at RT at the indicated concentrations: anti-RAI1 1:200 (rabbit, 58658 Abcam), anti-LC3 1:300 (rabbit, L7543 Sigma), Alexa Fluor-488 1:500 (Jackson ImmunoResearch, West Grove, PA, USA). For nuclear staining, the cells were incubated with Hoechst 33342 (trihydrochloride, trihydrate, Thermo Fisher Scientific) diluted 1:10000 in 1X PBS for 5 min. All immunofluorescence images were acquired using a Nikon C2 microscope with 20X and 60X objectives and the NIS Elements 1.49 program. The same camera settings were used for the replicates. Adobe Photoshop 7.0 software was used to merge the images.

### RAI1 mutants generation

Vectors expressing RAI1 mutants (s3999p40fx-HA, s3999p40fx-flag, Q214x-HA and Q214x-flag) were generated by PCR using high proofreading Q5 polymerase (M0491S, New England Biolabs). The restriction enzyme sites for SalI and NotI were added into the primers for inserts amplification and used to digest the fragments. The digested PCR products were ligated with the pAlter vector digested with SalI and NotI using the T4 DNA ligase (Biolabs, M0202). The ratio plasmid: insert used was from 1:3 up to 1:10 according to their length in base pairs. The reaction was used to transform TOP10 E.Coli according to a standard transformation protocol. All plasmids were finally controlled by Sanger sequencing.

Primers list (for PCR)

RAI1 wt For

5′ CCCGGGGTCGACCCACGCGTCCGC 3′

RAI1-S399P40fx-HA Rev

5′CGTTAAGCGGCCGCTTAAGCGTAATCTGGAACATCGTATGGGTAGCGCCGTGAGGC 3′

RAI1-S399P40fx-flag Rev

5′CGTTAAGCGGCCGCTTACTTATCGTCGTCATCCTTGTAATCGCGCCGTGAGGC 3′

RAI1-Q214stop-HA Rev

5′cgttaaGCGGCCGCctaAGCGTAATCTGGAACATCGTATGGGTAggaatgctgagg 3′

RAI1-Q214stop-flag Rev

5′cgttaaGCGGCCGCctaCTTATCGTCGTCATCCTTGTAATCggaatgctgagg 3′

### HEK293T transfection

HEK293T cells were transfected with polyethyleneimine (PEI) linear MW 25,000 Da (Sigma-Aldrich) according to the dimensions of the well. DNA: PEI (0.5% v/v) ratio was 2:1.

### Microarray analysis

GeneChip Human Transcriptome Array (HTA) 2.0 was used for the quantification of transcript levels. All procedures were conducted according to the manufacturer’s protocols (Affymetrix Inc., Santa Clara, CA, USA). Briefly, for each array, complementary RNA (cRNA) was prepared from 200 ng of total RNA according to the Affymetrix Whole Transcript (WT) protocol. The cRNA was used to generate single-stranded DNA, which was fragmented and biotinylated. The labeled single-stranded DNA was hybridized on Affymetrix HTA 2.0 microarrays. The microarrays were then washed and stained with a streptavidin-phycoerythrin conjugate in an Affymetrix Fluidics Station 450. The microarrays were scanned with a GeneChip Scanner 3000 7 G (Affymetrix). All GeneChips were visually inspected for irregularities. The global method of scaling (or normalization) was applied to all GeneChips. Various quality measures, such as the percentage of present genes and the ratio of endogenous genes, indicated a high overall quality of samples and assays. Scanning and data extraction of the microarray were followed by transformation of the fluorescence data into CEL files using Affymetrix GeneChip Command Console (AGCC) software. The Simpleaffy package (v. 2.66) from Bioconductor was used for the extraction of quality measurements from the raw CEL files and for RMA normalization [16]. In the process, 5% of the less variable probsets among the samples (3525 probsets) was removed. Differential gene expression analysis of protein-coding genes was performed using the limma package (v.3.46.0) [17]. Differentially expressed genes were considered significant if their *p* value after Bonferroni correction was <0.05. The enrichment of GO terms was evaluated using Fisher’s exact test, and in this process, the background set consisted of all coding genes detected in either RAI1-S399P40fx or sibling cells [18]. GO terms were considered significantly enriched if their *p* value after Bonferroni correction was <0.05. The ArrayExpress accession number was E-MTAB-10118.

### RNA extraction and reverse transcription

Total RNA was isolated using TRIzol reagent (Life Technologies) according to the manufacturer’s instructions. The RNA quality was assessed by determining the UV absorbance at 260 nm using a Qubit 3.0 fluorometer (Thermo Scientific). The RNA size distribution was analyzed using RNA 6000 Nano LabChips (Agilent Technologies) and processed using the Agilent 2100 Bioanalyzer with the total RNA electrophoresis program. All RNAs were treated with DNase before reverse transcription. Only RNAs with an RNA integrity number (RIN) ≥ 8 were used for subsequent analysis. Reverse transcription was performed using a High Capacity cDNA Reverse Transcription Kit (Applied Biosystems) according to the manufacturer’s instructions after digestion with DNase I (Life Technologies).

### Quantitative real-time polymerase chain reaction (qRT‒PCR)

Real-time PCR was performed using a 7900HT Fast Real-Time PCR system (Applied Biosystems). SYBR Green reactions were performed using the Power SYBR Green PCR Master Mix (Applied Biosystem) with the following program: denaturation at 95 °C for 10 min; 40 cycles of amplification at 95 °C for 10 s, 60 °C for 10 s, and 72 °C for 30 s; final elongation at 72 °C for 7 min; and final dissociation at 95 °C for 15 s, 60 °C for 15 s, and 95 °C for 15 s. For each gene of interest, qRT‒PCR was performed as follows: each RNA sample was tested in duplicate, and 18 S or TBP was used as a reference. The 2–ΔΔCT method was used for the calculation of relative expression levels. Statistical analyses of data from three independent experiments were performed.

The primers used in the study are listed in the following table.

**Table:** 

Primer ID	Forward primer	Reverse primer
RAI1	CCC AGG AGC ACT GGG TGC ATG A	GCAGCTGGAACACATCATGTCCAC G
SQLE	CTTCTCCTCAAAGCGAGCACA	TTATTTAAAAATCGCCTGCTGGA
DHCR7	ACAGAACCGCATCTCAAGGG	CTG TAC TGG TCA CAA GCC
ASAH1	GCACAAGTTATGAAGGAAGCCAAG	TCCAATGATTCCTTTCTGTCTCG
HEXA	GTCATTGAATACGCACGGC	G GCT CAG ACC CAG AGT AG
HEXB	CCG TGA TCT CCG CAC CGA	CCG TGA CCG TCG CTA GAA C
LGMN	GCATAGGATCCGGCAAAGTC	TCCAGTAGATCCA TGGTCAGTGA
CTNS	TTCGTGGCTCTGAACCTGAC	A TCA GCG TGA GGA CAA CC
IGF2R	CTCTAAGTGCGGAAAGGATA	TCCATCAGCCACTCTGTTT
SLC17A5	CGGGTAAGAAGTACCAATGGG	CCTCCAGGAATCTGTGTGATG
SORT1	GGGGACACATGGAGCATGG	GGAATAGACAATGCCTCGATCAT
CLN3	CTCTGTCTCTACGGCTGCTG	TAGCGAAGACCACACCACAC
CTSH	GTACTGGTCCCTACCCACC	TTCCGCCAAGGACAGCATC
CDA	CAGTCACTTTCCTGTGGGGG	CATCTTGCATGTCACTGGCG
TGFB2	CTTGTGCTCCAGACAGTCCC	AGGAGAGCCATTCGCCTTC
MT2A	CTAGCCGCCTCTTCAGCTC	AAGTCGCGTTCTTTACATCTGG
MT1A	GCTCGAAATGGACCCCAAC	TAAATGGGTCAGGGTTGTATGG
MT1M	AGCAGTCGCTCCATTTATCG	GCTCTTCTTGCAGGAGGTG
MT1G	CTAGTCTCGCCTCGGGTTG	GCAGCTGCACTTCTCCGAT
MT1E	GCTTGTTCGTCTCACTGGTG	CAGGTTGTGCAGGTTGTTCTA
18 s	CAGGATTGACAGATTGATAGCTCTTTC	ATCGCTCCACCAACTAAGAAC
β-Actin	GGCATCCTCACCCTGAAGTA	GGGGTGTTGAAGGTCTCAAA

### Untargeted lipidomics analysis

Lipids were extracted using a methanol:MTBE:chloroform (MMC) mixture (40/30/30, v/v/v) [19] containing 1 mg/L 2,6-di-butyl-p-hydroxytoluene (BHT) as an antioxidant and an isotopically labeled internal standard mix (EquiSPLASH LIPIDOMIX Quantitative Mass Spec Internal Standard code). 330731, Avanti Polar Lipids) at a concentration of 2.5 µg/mL. Labetalol (1 µM) was used as an injection standard. The samples were normalized to the cell number during the extraction phase by using an appropriate volume of mixture (1 mL/2.5 × 10^6^ cells). Technical quality controls (TQCs) were run before and during sample batch acquisition to assess the instrument performance. EquiSPLASH Lipidomix Quantitative Mass Spec Internal Standard diluted to 2.5 µg/mL was used for the TQC. The samples were then well vortexed and shaken at room temperature for 30 min (950 rpm). Afterward, the samples were centrifuged for 10 min at 8000 rpm. The supernatants were collected in fresh tubes, and 2 μL was used for the LC‒MS analysis. The LC‒MS system consisted of a Dionex UltiMate 3000 series (Thermo Fisher Scientific, Waltham, MA, USA) with a binary pump, a thermostated autosampler, a column compartment, and a Thermo Q-exactive mass spectrometer (Thermo Fisher Scientific, Waltham, MA, USA). Liquid chromatography separation was performed at 45 °C using a Kinetex F5 reverse-phase column (Phenomenex Inc.) at a flow rate of 0.65 mL/minutes. The mobile phases consisted of 5 mM ammonium formate and 0.1% formic acid in water (solvent A) and 5 mM ammonium formate and 0.1% formic acid in isopropanol (solvent B). Gradient elution was used for lipid separation as follows: 0 min, 80% solvent A, 20% solvent B; 3 min, 60% solvent A, 40% solvent B; 16 min, 40% solvent A, 60% solvent B; 16.5 min, 30% solvent A, 70% solvent B; 24 min, 26% solvent A, 74% solvent B; 28 min, 5% solvent A, 95% solvent B; and 30 min, termination of the program. All solvents were purchased from Sigma‒Aldrich. Mass spectrometry analysis was first performed using the positive/negative ion switching method in the full MS scan mode. The obtained high-resolution mass list was then processed using Lipostar software (version 1.3.0, Molecular Discovery Ltd., UK) for the preidentification (by mass searching within a library of approximately 800,000 in silico fragmented lipids) of potential lipid species based on the m/z and retention time values. The masses of interest generated after this preidentification step were used for the Inclusion Lists applied to reanalyse a reduced number of samples automatically selected by Lipostar in the DDA mode to obtain MS/MS data. The MS/MS data were then imported into the data matrix generated by Lipostar to perform the final lipid identification step based on the exact mass, retention time and MS/MS fragmentation. Automatically generated data were visually inspected, and only high-confidence data were selected for the statistical analysis. Lipostar software was used for the multivariate statistical analysis (PCA and O-PLS-DA), and Pareto scaling was applied. For the PCA model, the first two principal components (PC1 and PC2) accounted for 56% of the explained variance. In the O-PLS-DA model, the classes consisted of the control and Smith-Magenis samples. The OPLS-DA showed perfect fitting and high predictability of the model with high statistical values of R2 (1) and Q2 (0.91) at the first latent variable (LV).

### Western blot analysis

Approximately 1 × 10^6^ cells were plated 24 h before collection. For chloroquine treatment, cellular pellets were lysed with RIPA buffer (5 M NaClO, 25 M Na_2_HPO_4_, 0.5 M NaH_2_PO_0_, 0.5 M EDTA pH 8, 10% NaDOC, 10% Triton X-100, and 10% SDS), and 1:100 protease inhibitors were added before use. The lysed cells were maintained on ice for 20 min and then sonicated for 10 s continuously at an output of 2. After centrifugation for 10 min at 4 °C and 13,000 rpm, the supernatant was collected, quantified and stored at −80 °C. The protein concentration was measured using the bicinchoninic acid assay method. Equal amounts of proteins were separated by 12% Tris-HCl SDS‒PAGE. The following primary antibodies were used: β-tubulin (T7816, 1:5000), LC3B (L7543, 1:1000), and p62 (P0067, 1:1000). Immobilon® Classico Western HRP substrate (Millipore) was used to reveal proteins. Chemiluminescent detection was performed on an LAS-4000 mini instrument (Fujifilm, Düsseldorf, Germany). The quantifications were performed using ImageJ 1.45 software.

### Electron microscopy

The samples were fixed with 2.5% glutaraldehyde in 0.1 M sodium cacodylate buffer pH 7.4 overnight at 4 °C. The samples were postfixed with 1% osmium tetroxide plus 1% potassium ferrocyanide in 0.1 M sodium cacodylate buffer for 1 h at 4 °C. After three washes with water, the samples were dehydrated through a graded ethanol series and embedded in epoxy resin (Sigma‒Aldrich). Ultrathin sections (60–70 nm) were obtained with an Ultrotome V (LKB) ultramicrotome, counterstained with uranyl acetate and lead citrate and viewed with a Tecnai G2 (FEI) transmission electron microscope operating at 100 kV. Images were captured with a Veleta (Olympus Soft Imaging System) digital camera.

### Electron microscopy image analysis

To evaluate the percentage of the cytoplasmic area occupied by empty vesicles (EVs) and the number of EVs, 10 images (scale bar: 5 μm) from three different biological replicates were processed using ImageJ software by subtracting the sum of the areas of the individual vesicles from the total cytoplasmic area (whole cell-nucleus). The expansion of the endoplasmic reticulum (ER) was quantified by the ratio of the ER to nucleus areas, whereas the number of swollen mitochondria (Smt) was manually counted from 10 image fields (scale bar 500 nm) of six cells obtained from three different experiments (60 images total).

### Production of lentiviral particles and infection

LV-mitoKeima was kindly provided by Toren Finkel (Center for Molecular Medicine, National Heart, Lung, and Blood Institute, NIH, Bethesda, MD, USA). HEK293 cells were seeded onto 100-mm Petri dishes. Twenty-four hours after plating, the cells were cotransfected using PEI with LV-mitoKeima and the packaging plasmids pMDLg/pRRE, pRSV-Rev, and pMD2.G. After 8 h, the transfection medium was replaced with fresh culture medium. Two million MEFs were seeded onto 60-mm Petri dishes. After 48 h, the cell medium was collected and mixed with 6 μg/mL polybrene. MEFs were infected for 36 h before the medium was changed. All procedures related to the production and use of lentiviral particles were performed in a biosafety level-2 environment.

### MitoTracker mitophagy analysis by flow cytometry

MitoKeima-expressing fibroblasts were analyzed as previously reported (Balsalobre et al., 1998; Um et al., 2018). Cells were analyzed using a flow cytometer (BD FACSAriaTM sorter) equipped with 405-nm and 561-nm lasers. The cells were simultaneously excited with a violet laser (405 nm) and a yellow‒green laser (561 nm), and the emission was detected at 610 ± 10 nm with a BV605 detector and at 610 ± 10 nm by a PE-437 CF594 detector, respectively.

### Cell death percentage

Approximately 1 × 10^5^ fibroblasts were seeded in a 6-well plate at the same concentration per well. Cell counts were performed in a blind manner using the Bürker Chamber. Trypan blue was added to the cell suspension (Sigma‒Aldrich, 5 μL each; dilution factor 1:2) and then introduced into the device by capillary action. The percentage of dead cells was calculated by dividing the number of Trypan blue-positive cells by the total number of cells (dead cells plus live cells).

### Reactive oxygen species (ROS) quantification

Cell ROS generation was assessed using the fluorescent probe 2´,7´-dichlorodihydrofluorescein diacetate (DCFDA). Approximately 8 × 10^5^/ml fibroblasts were seeded in 24-well (black) plates. The next day, the cells were washed twice with 1X PBS with Ca^2+^ and Mg^2+^ and then incubated with 10 μM DCFDA. The fluorescence emission at 520 nm was monitored for 120 min using a VICTOR™ Multilabel Counter, PerkinElmer. The fluorescence emission at 60 min after dye loading is reported as ROS production.

### Terminal deoxynucleotide transferase-mediated dUTP nick end-labeling assay (TUNEL)

The percentage of apoptotic cells was determined by TUNEL assay using an In Situ Cell Death Detection Kit (Roche Diagnostics GmbH, Mannheim, Germany). Approximately 40,000 cells were plated in Cultrex® Basement Membrane Matrix (Trevigen)-pretreated coverglasses. The cells were fixed in 4% paraformaldehyde/PBS for 10 min and washed for 5 min with 1X PBS with Ca^2+^ and Mg^2+^. The cells were then permeabilized with 0.1% Triton X-100 (Sigma Aldrich Company, St. Louis, MO, USA) at 4 °C for 5 min and then incubated with the TUNEL reaction mixture (50 μL) in a humidified chamber in a dark room at 37 °C for 1 h. For nuclear staining, the cells were incubated with Hoechst 33342 (Trihydrochloride, Trihydrate − 10 mg/mL Solution in water, Thermo Fisher Scientific) diluted 1:10000 in 1X PBS for 5 min. The samples were analyzed under a Nikon C2 microscope with 40X objectives and the NIS Elements 1.49 program. The TUNEL-positive cell percentage was calculated as follows from three independent experiments: number of TUNEL-positive nuclei/number of DAPI-stained cells × 100%.

### Oil Red O (ORO) staining and analysis

Approximately 5000 cells were plated in a 96-well dish with 12 replicates per line, and approximately 30,000 cells were plated in a 145-μm Cell Imaging Dish with three different biological replicates. Twenty-four hours after plating, the cells were washed with 1X PBS, fixed with 4% paraformaldehyde for 10 min, washed once with dH_2_O and incubated in 60% isopropanol for 5 min. The isopropanol was then removed, and the cells were incubated in Oil Red O (ORO) staining solution (0.3% ORO in 60% isopropanol) for 10 min and washed five times with dH_2_O. For quantification of the absorbance of cells in the 96-well dish, ORO was extracted using 100% isopropanol (50 μL/well) for 10 min, and absorbances were measured (490 nm). The measured values were normalized to the protein concentration per well. To count the number of LDs per cell in the Cell Imaging Dish, after ORO staining, the dish was washed with PBS and incubated for 5 min with Hoechst 33342 (Trihydrochloride, Trihydrate, Thermo Fisher Scientific) diluted 1:10000 in 1X PBS for nuclear staining. Images of 10 fields of each replicate were captured with a Nikon C2 microscope with 20X objectives and NIS Elements 1.49 program. The number of LDs was quantified using ImageJ 1.45 software.

### BODIPY 493/503 staining

Approximately 30.000 cells were plated in a 145-μm Cell Imaging Dish. After 24 h, the cells were washed with 1X PBS and then incubated with BODIPY 493/503 staining solution (10 μM BODIPY 493/503 in PBS) for 15 min at 37 °C. After incubation, the cell nuclei were stained with Hoechst 33342 (Trihydrochloride, Trihydrate, Thermo Fisher Scientific) diluted 1:10000 in 1X PBS for 5 min. The samples were analyzed under a Nikon C2 microscope with 40X objectives and the NIS Elements 1.49 program. The size and number of LDs were quantified using ImageJ 1.45 software.

### LysoTracker^TM^ Red DND-99 staining

Approximately 30,000 cells were plated in a 145-μm Cell Imaging Dish, and after 24 h, the cells were incubated with LysoTracker™ Red DND-99 staining solution (70 µM LysoTracker™ Red DND-99 in fresh medium) for 90 min at 37 °C. After incubation, the cell nuclei were stained with Hoechst 33342 (trihydrochloride, trihydrate, Thermo Fisher Scientific) diluted 1:10000 in 1X PBS for 5 min. The samples were analyzed under a Nikon C2 microscope with 40X objectives and the NIS Elements 1.49 program. The corrected total cell fluorescence (CTCF) was quantified using ImageJ 1.45 software.

### Statistics

Cell analyses were performed with at least three independent experiments in duplicate or triplicate (unless indicated), and imaging experiments were quantified using different (>5) fields of view. Statistical analyses were performed using GraphPad Prism 7 software (GraphPad software, v 7.01, San Diego, CA, USA) or R (v. 4.0.3) (www.R-project.org) by either two-tailed Student’s t test or two-way ANOVA followed by Newman‒Keuls test. If more than one statistical test was performed, the p values were corrected for multiple testing using the Bonferroni method. Assumptions concerning the data (for example, normal distribution and similar variation between experimental groups) were examined for appropriateness before the statistical tests were conducted. Unless otherwise specified, the data are shown as the means ± standard errors of the mean (SEMs). Differences were statistically significant if the p value, after correction in the case of multiple tests, was lower than 0.05: *p < 0.05, **p < 0.01, and ***p < 0.001.

## Results

### RAI1 is reduced in SMS patient-derived cells and localizes mainly to the cytosol

**Fig. 1:**
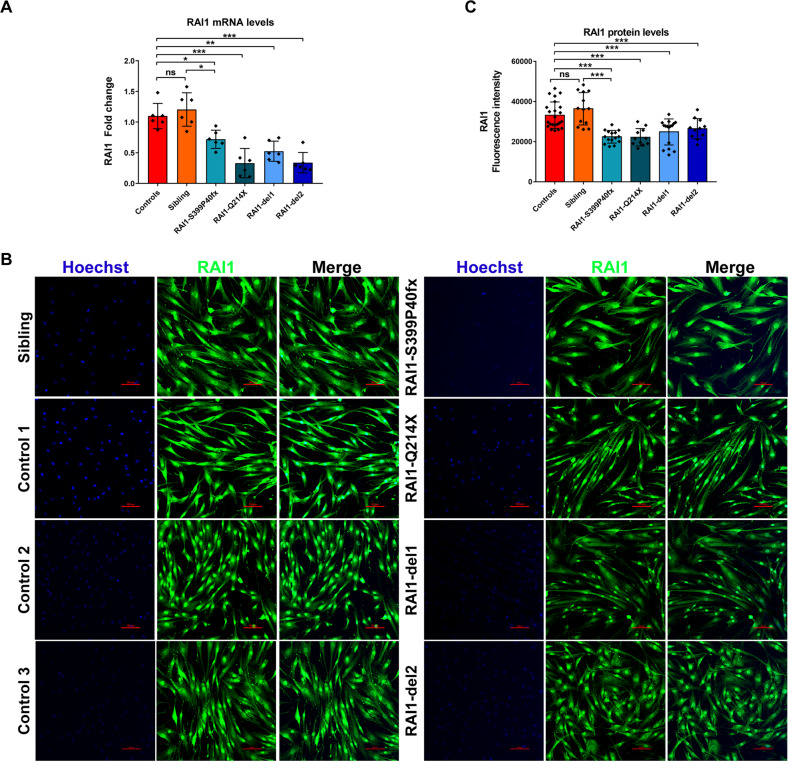
Subcellular localization of RAI1 in SMS patient-derived cells. **A** The RT–PCR analysis of the transcript levels of *RAI1* revealed reduced gene expression in SMS cells compared with control cells (*n* = 6–7 biological replicates). **B** An immunofluorescence analysis of the subcellular localization of RAI1 in SMS and control cells revealed that wild-type and mutant RAI1 localize to the nucleus and cytosol. RAI1 (green) was detected using a specific antibody, and nuclei were detected with Hoechst (blue) (*n* = 3 biological replicates). Scale bar = 25 µm. Representative images are shown. **C** Densitometry showing the RAI1 levels (*n* = 15 cells from 5 randomly selected fields from 3 independent experiments of each cell line). Graphs: mean ± SEM, one-way ANOVA + Newman–Keuls post hoc test, *p < 0.05, **p < 0.01, ***p < 0.001, ns not significant.

To shed light on the mechanisms through which *RAI1* haploinsufficiency causes disease, we collected skin biopsies from two heterozygous female SMS patients carrying two CNVs, one with the canonical deletion of 3.6 Mb, which includes 72 genes (hereafter referred to as RAI1-del1), and the other with a 1-Mb deletion that includes 2 genes, *FLCN* (folliculin) and *RAI1* (RAI1-del2). Moreover, we obtained biopsies from two other female heterozygous SMS patients with different *RAI1* point mutations. One patient carries a de novo 1-bp deletion (NM_030665.3:c.1194delC) resulting in a serine 399-to-proline substitution (S399P) followed by a 40-amino-acid frameshift terminated by a premature stop codon (hereafter referred to as RAI1-S399P40fx), previously identified in an Italian family [20]. This patient also carries a familial duplication of the cholinergic receptor nicotinic alpha 7 subunit (*CHRNA7*) gene. The other patient carries a nucleotide substitution (NM_030665:c. [640 C > T]), which results in the conversion of glutamine 214 (Q214) to a premature stop codon (hereafter referred to as RAI1-Q214X). This patient also carries a second mutation in the gene encoding methyl-CpG binding domain protein 5 (*MBD5*, NM_018328:c. [3691 C > A]), resulting in a proline 1231-to-threonine substitution. Importantly, this gene is associated with an SMS-like phenotype [21]. As controls (CTRs), we used fibroblasts derived from three healthy unrelated subjects (hereafter referred to as CTR1, CTR2, and CTR3). Because the RAI1-S399P40fx patient carries the CHRNA7 duplication, as a control, we obtained a skin biopsy specimen from her healthy sister, who carries the same duplication but is negative for the SMS mutation (sibling). To reduce variability, all the biopsies (SMS and controls) were obtained from the same site of the arm through a standardized procedure, and all experiments were performed using cells cultured for no more than 11 passages to avoid senescence. In each different experimental setup, we used cells at the same passage number. We first analyzed the expression levels of RAI1 and found that the transcript levels of the *RAI1* gene were decreased by 30–60% in the SMS cells compared with the control cells (Fig. 1A). We then analyzed the RAI1 protein levels in our cells. To this end, we used an anti-RAI1 antibody that specifically recognizes full-length and mutant (RAI1-S399P40fx and RAI1-Q214X) RAI1 in HEK293T cells (Supplementary Fig. 1). Although we could not detect endogenous RAI1 in fibroblast cell lines by Western blot analysis using this antibody, we detected the RAI1 protein levels and subcellular localization by immunofluorescence and confocal microscopy (Fig. 1B). A densitometric analysis revealed that the intensity of RAI1 staining was decreased by approximately 30–40% in the SMS cells compared with the control cells (Fig. 1C). In addition, we found that RAI1 localized to both the nucleus and cytosol. These results indicate that SMS cells express lower levels of RAI1 than control cells.

### SMS patient-derived cells show deregulated expression of genes involved in lipid metabolism, lysosome function, and protein/lipid trafficking

**Fig. 2:**
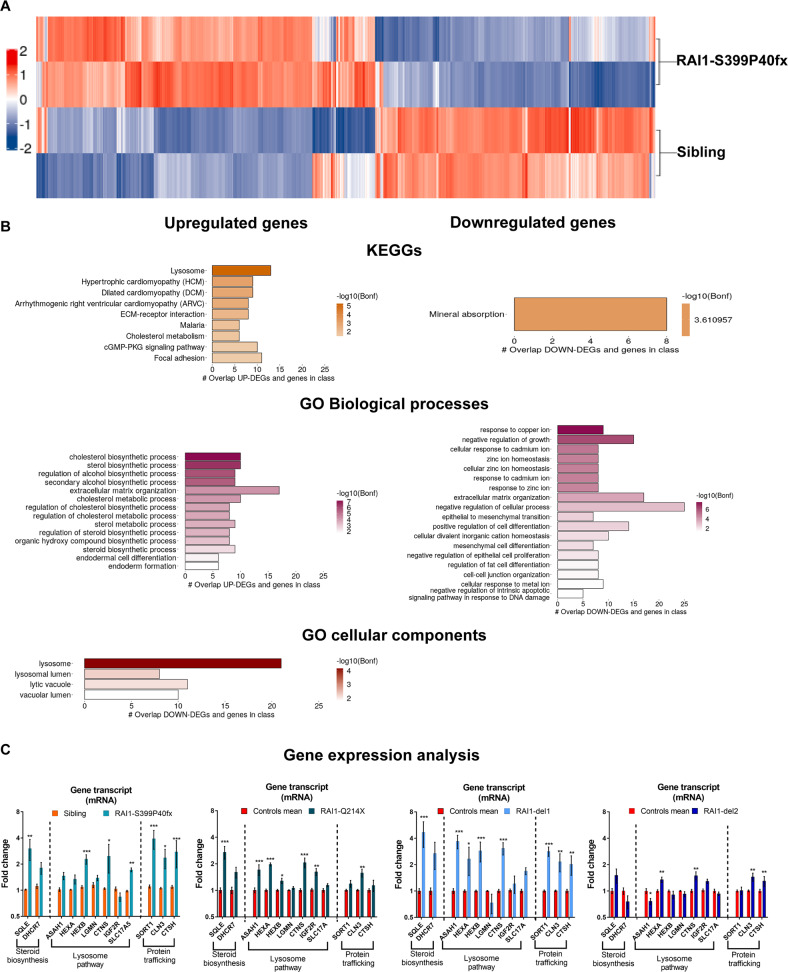
RAI1 haploinsufficiency alters the expression of genes involved in lipid metabolism, lysosome function and protein/lipid trafficking. **A** Heatmap of the 25% most variable coding genes (*n* = 6183) identified from the whole-genome microarray analysis of RAI1-S399P40fx and sibling cells. The *Z* scores of gene expression are depicted as a gradient from blue (low expression) to red (high expression) (*n* = 2 biological replicates). **B** Kyoto Encyclopedia of Genes and Genomes (KEGG) and Gene Ontology (GO) analysis of DEGs in RAI1-S399P40fx cells relative to sibling cells. **C** Quantitative real-time PCR of selected genes in four SMS cell lines and four control cell lines (*n* = 5 biological replicates). Control = average CTR1/2/3. Graphs: mean ± SEM, one-way ANOVA + Newman–Keuls post hoc test, *p < 0.05, **p < 0.01, ***p < 0.001.

To investigate the pathological processes occurring in SMS, we took an unbiased approach and performed a whole-genome microarray analysis to identify the differentially expressed genes (DEGs) associated with *RAI1* haploinsufficiency. To avoid the confounding effects of the loss of multiple genes in SMS-del cells, we used RAI1-S399P40fx cells and sibling cells. We analyzed total mRNA from two replicates of each cell line. We identified 638 differentially expressed probes (*p* value after Bonferroni correction ≤0.05) corresponding to 558 DEGs, and 265 and 293 of these DEGs were significantly upregulated (from 303 upregulated probsets) and downregulated (from 335 downregulated probsets) in the SMS cells, respectively (Fig. 2A, Supplementary Table 1). Kyoto Encyclopedia of Genes and Genomes (KEGG) and Gene Ontology (GO) analyses based on “Biological process” and “Cellular component” terms revealed that the top significantly enriched pathways were “*steroid biosynthesis*”, “*lysosome*”, and “*mineral absorption*” (Fig. 2B). We then selected upregulated and downregulated genes for validation using RAI1-S399P40fx cells via RT‒PCR. In addition, we performed a gene expression analysis of RAI1-Q214X, RAI1-del1, and RAI1-del2 cell lines (Fig. 2C). Considering the variability intrinsic to primary cells derived from patients, we confirmed that the mRNA transcript levels of genes involved in steroid biosynthesis, namely, squalene epoxidase (*SQLE*) and 7-dehydrocholesterol reductase (*DHCR7*) [22, 23]; the lysosome pathway, including N-acyl sphingosine amidohydrolase 1 (*ASAH1*), hexosaminidase subunits alpha and beta (*HEXA* and *HEXB*), legumain (LGMN), cystinosin (*CTNS*), insulin-like growth factor 2 receptor (*IGF2R*), and sialin (solute carrier family 17 member 5, *SLC17A5*); protein trafficking, including sortilin 1 (*SORT1*) and battenin (*CLN3*); and cathepsin H (*CTSH*), were upregulated in most lines. We did not find any expression changes for any of the downregulated genes in our SMS cells (Supplementary Fig. 2). These results show that RAI1 haploinsufficiency directly or indirectly influences the expression of genes encoding enzymes involved in the steroid biosynthetic pathway, lysosomal pathway, and protein trafficking.

### *RAI1* haploinsufficiency alters lipid metabolism

**Fig. 3:**
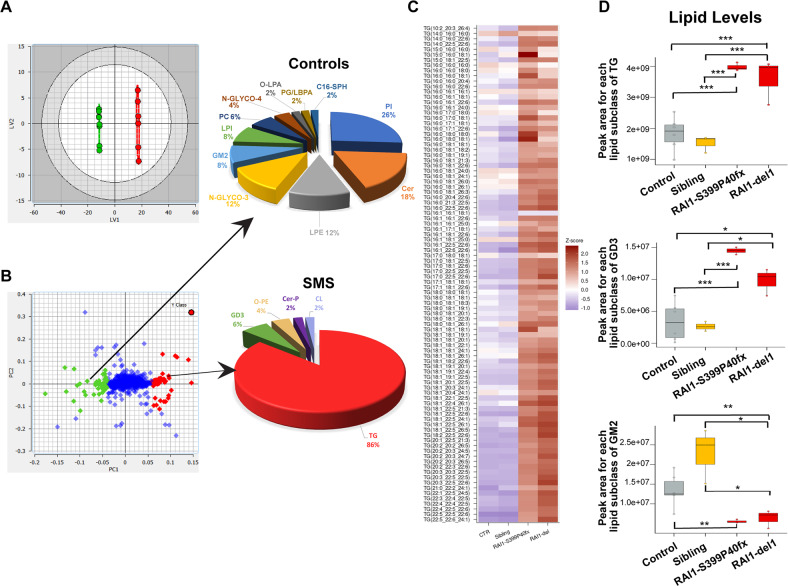
*RAI1* haploinsufficiency alters the lipid profile. **A** O-PLS-DA LV1-LV2 score plot of untargeted lipidomic analysis revealed distinct lipid profiles between SMS cells (RAI1-del1 and RAI1-S399P40fx, green) and CTRL (CTR1/2 and sibling, red) cells (*n* = 3 biological replicates). **B** O-PLS-DA LV1-LV2 weight plot. The 100 most discriminant lipids are colored red (higher in SMS samples) and green (higher in control samples). The compositions of the 50 most representative lipids in the control and SMS samples are shown. **C** Heatmap of triglycerides. **D** Box plots showing the relative TG and GM2/GD3 ganglioside abundances in control, SMS and CTR cells. The relative abundance was derived by adding the peak area for each lipid subclass identified by LC–MS and Lipostar software. The boxplots show the medians (horizontal lines), 25th–75th percentiles (box outlines), and highest and lowest values within 1.5x of the interquartile range (vertical lines). Graphs: mean ± SEM, one-way ANOVA + Newman–Keuls post hoc test, *p < 0.05, **p < 0.01, ***p < 0.001.

We conducted an untargeted lipidomic analysis to identify specific lipid changes associated with the disease. We compared the global lipid profile of two SMS patient cell lines (RAI1-S399P40fx and RAI1-del1), which are representative of the two types of mutations causative of SMS, with that of healthy controls (CTR1, CTR2, and sibling cells). After synchronization through dexamethasone, we collected three biological replicates of each cell line and processed them for LC–MS/MS untargeted lipidomic analysis using Lipostar software [24]. We identified 730 lipid species, which belonged to four lipid categories (sterols, sphingolipids, glycerophospholipids, and glycerolipids) and approximately 30 subclasses (Supplementary Table 2–3). All these species were present in both healthy (728/730) and SMS (726/730) cells. To characterize the differences, principal component analysis (PCA) [25] and orthogonal projections to latent structures-discriminant analysis (OPLS-DA) plots were generated. We found that the SMS and control samples clustered into different groups. Of note, the sibling cells clustered together with the CTR1 and CTR2 cells, indicating a similar lipidomic profile (Fig. 3A). Using the weight plot, we separated the lipid species enriched in either the SMS cluster (red) or the control cluster (green) and the lipids enriched in both the control and SMS groups (blue) (Fig. 3B). We then analyzed the 50 lipids most enriched among the SMS cells (those within the red cluster) and the 50 lipids most enriched among the control cells (those within the green cluster) (Fig. 3B, Supplementary Table 4). We found that SMS cells had significantly increased levels of triacylglycerols (TGs), corresponding to 86% of these 50 lipids, compared with control cells (Fig. 3B, C), whereas in the control cells, diacylglycerophosphoinositols were the most represented class (26%) among the 50 most discriminant lipids, followed by ceramides (Cers, 18%), monoacylglycerophosphoethanolamines (12%) and gangliosides (12%) (Fig. 3B, Supplementary Table 4).

We also found decreased levels of Cers and neutral glycosphingolipids with two, three, and four sugar units as well as alterations of the ganglioside pathway [26], as revealed by increased levels of GD3 species and decreased levels of GM2 species in SMS cells compared with control cells. After combining CTR1 and CTR2, a box plot analysis showed significantly increased levels of TGs (RAI1-S399P40fx_vs_CTRs, *p* = 0.00031, FC = 2.17; RAI1-del1_vs_CTRs, *p* = 0.004, FC = 1.96, RAI1-S399P40fx_vs_Sibling, *p* = 0.00014, FC = 2.61; RAI1-del1_vs_Sibling, *p* = 0.0097, FC = 2.36) and GD3 (RAI1-S399P40fx_vs_CTRs, *p* = 0.00047, FC = 4.27; RAI1-del1_vs_CTRs, *p* = 0.013, FC = 2.88, RAI1-S399P40fx_vs_Sibling, *p* = 2.7e-05, FC = 5.49; RAI1-del1_vs_Sibling, *p* = 0.005, FC:3.71) and decreased levels of GM2 (RAI1-S399P40fx_vs_CTRs, *p* = 0.014, FC = 0.41; RAI1-del1_vs_CTRs, *p* = 0.029, FC = 0.47, RAI1-S399P40fx_vs_Sibling, *p* = 0.012, FC = 0.24; RAI1-del1_vs_Sibling, *p* = 0.017, FC = 0.27) (Fig. 3D). The GM2 lipid species were different among the control and sibling cells, likely as a result of the variability among genetic backgrounds, but this difference was not significant. Together, these observations show that RAI1 haploinsufficiency alters the levels of TGs, sphingolipids, Cers, and gangliosides and results in the accumulation of TGs.

### Increased LD accumulation in SMS cells

**Fig. 4:**
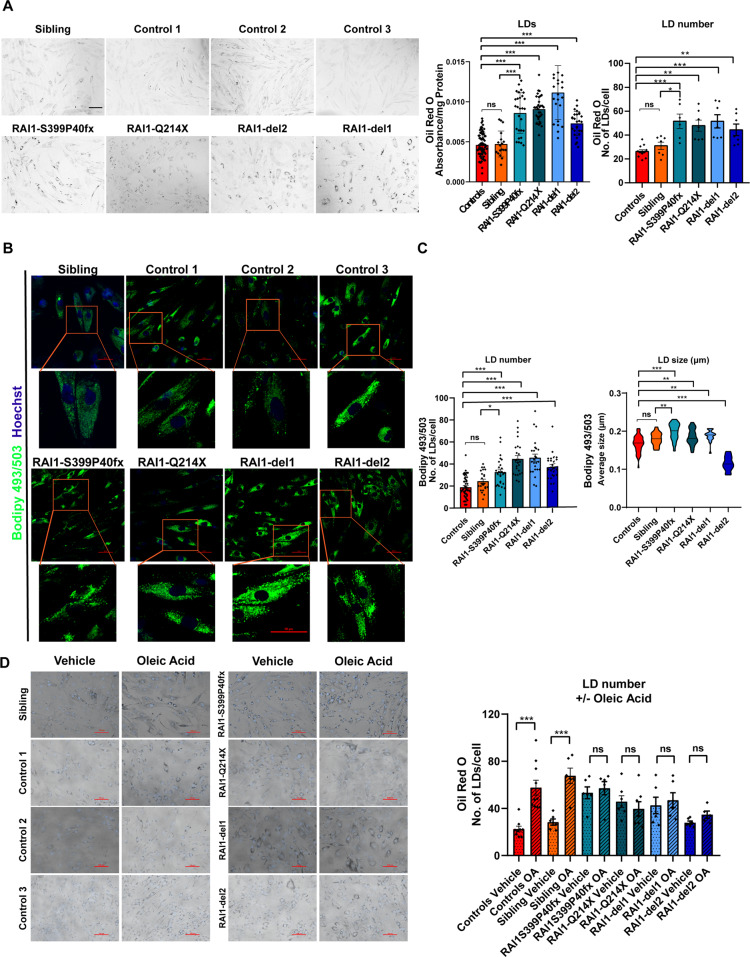
Accumulation of lipid droplets in SMS patient-derived cells. **A** Left panel: Representative fluorescence microscopy images of lipid droplets (LDs) colored with Oil Red O in SMS and CTR cells (*n* = 5 biological replicates). Middle panel: Quantification of the optical density of nine independent wells from five biological replicates. Right panel: Quantification of the number of LDs per cell among 150 cells from three biological replicates. **B**, **C** BODIPY staining analysis of the number and size of LDs in SMS and CTR cells. Left panel: Representative images of 10 fields from three biological replicates. Middle panel: Quantification of the LD number of 10 cells from 10 randomly selected fields from three independent experiments of each cell line. Right panel: Quantification of the size of LDs per cell among 10 cells from 10 randomly selected fields from three independent experiments of each cell line. **D** Oil Red O staining in four SMS and four CTR cell lines treated with vehicle and oleic acid (OA, 50 μM, 48 h). Left panel: Representative images. Right panel: Quantification of the number of LDs per cell among 150 cells from three biological replicates. Graphs: mean ± SEM, one-way ANOVA + Newman–Keuls post hoc test, *p < 0.05, **p < 0.01, ***p < 0.001, ns not significant.

Eukaryotic cells store TGs in cytosolic LDs [27, 28]. To assess the potential accumulation of LDs in SMS cells, we stained all SMS and control cells with Oil Red O, a lysochrome fat-soluble diazo dye used for the staining of lipids, including TGs, and we quantified the amount of Oil Red O incorporated by the cells (Fig. 4A). We observed a significant 1.6- to 2-fold increase in Oil Red O staining in SMS cells compared with the control (CTR1, CTR2, CTR3) and sibling cells by either solubilizing the dye and measuring the optical density (Fig. 4A, left graph panel) or measuring the fluorescence signal for cell (Fig. 4A, right graph panel). Furthermore, we stained all SMS and CTR cells with the fluorescent dye BODIPY™ 493/503, which labels lipid-containing vesicles (Fig. 4B). We found that the number of LDs in each cell was increased by 1.5- to 2.5-fold in SMS cells compared with sibling and control cells (Fig. 4C, left graph panel). Interestingly, we also found that the size of LDs was significantly increased in all SMS cells, with the exception of RAI1-del2 (Fig. 4C, right graph panel). In RAI1-del2 cells, the number of LDs was increased, but their size was smaller compared with that in the control and other SMS cells. These findings show that cells carrying RAI1 haploinsufficiency accumulate LDs. The treatment of cells with Oleic Acid (OA) results in lipid accumulation and LD formation [29–31]. Thus, we treated the cells with 50 µM OA and stained them with Oil Red O (Fig. 4D). OA significantly increased lipid accumulation in control and sibling cells, as expected, but had no influence in SMS cells, which suggests that SMS cells cannot further produce LDs.

### Autophagy flux is defective in SMS cells

**Fig. 5:**
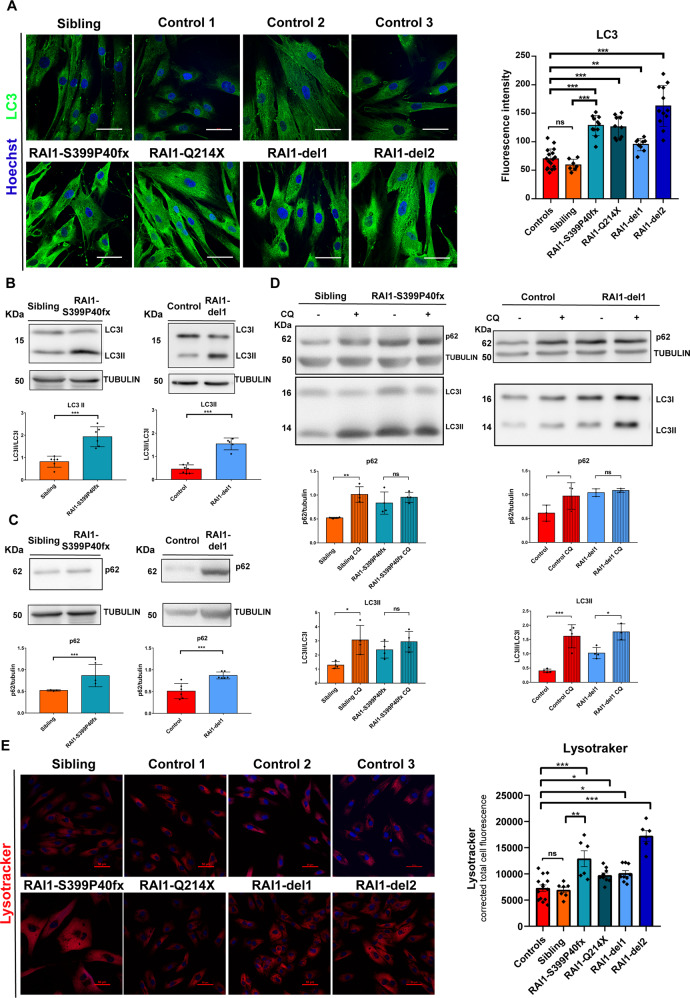
Aberrant autophagic flux in SMS patient-derived cells. **A** Immunofluorescence analysis of LC3 levels showing the accumulation of LC3 in SMS cells. Left panel: Representative images of *n* = 3 biological replicates. Right panel: Quantification of LC3 fluorescence intensity in 150 cells from three biological replicates. **B**, **C** Western blot analysis of the LC3 and p62 levels in SMS (RAI1-del1 and RAI1-S399P40fx) cells compared with sibling and CTR (sibling and CTR1) cells. Top panels: Representative images from three biological replicates. Bottom panels: Quantification of Western blots. **D** Western blotting analysis of the LC3 and p62 levels in SMS (RAI1-del1 and RAI1-S399P40fx) cells compared with sibling and CTR (sibling and CTR1) cells treated with either vehicle or chloroquine (CQ, 40 mM, 24 h). Top panels: Representative images from three biological replicates. Bottom panels: Quantification of Western blots. **E** LysoTracker staining analysis showing lysosomal accumulation in four SMS and four CTR cell lines. Left panel: Representative images of *n* = 3 biological replicates. Right panel: Quantification of LysoTracker fluorescence intensity in 100 cells from three biological replicates. Graphs: mean ± SEM, one-way ANOVA + Newman–Keuls post hoc test, *p < 0.05, **p < 0.01, ***p < 0.001.

The accumulation of LDs is often associated with defective autophagy [32–34]. We thus measured the levels of the autophagy marker lipidated LC3 (LC3II), which increases after the induction of autophagy [35], and sequestosome 1 (SQSTM1, p62), whose accumulation suggests aberrant autophagic flux [34]. By fluorescence analysis, we found that the LC3 levels were increased by approximately 1.4- to 2.2-fold in SMS cells compared with control cells (Fig. 5A). Western blotting revealed that the LC3II and p62 levels were increased by approximately 1.6- to 2-fold in RAI1-S399P40fx and RAI1-del1 cells compared with control cells (Fig. 5B, C). To test the existence of a block in autophagic flux in SMS cells, we treated the cells with chloroquine (CQ), which interferes with autophagosome-lysosome fusion and thus induces the accumulation of LC3II and p62 in cells with normal autophagic flux but not in cells with blocked autophagy [36]. CQ treatment resulted in the accumulation of LC3II and p62 in both CTR and sibling cells, as expected (Fig. 5D). CQ did not modify the p62 levels in SMS cells or the LC3II levels in RAI1-S399P40fx cells. CQ treatment increased the LC3II levels in RAI1-del1 cells but to a lesser extent than in control cells. Furthermore, staining of the cells with LysoTracker, which marks acidic organelles, revealed a significantly higher intensity signal in the SMS cells than in the control cells (Fig. 5E). These observations show that *RAI1* haploinsufficiency alters autophagic flux.

### Accumulation of vacuoles and swollen mitochondria in SMS cells

**Fig. 6:**
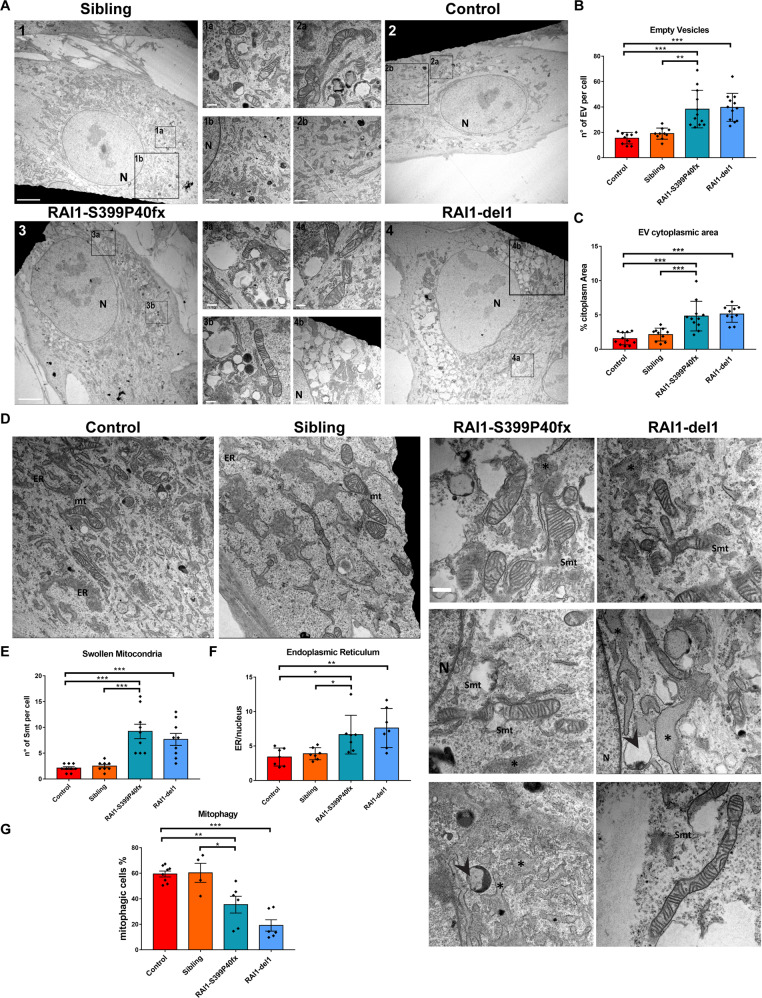
Accumulation of EVs, swollen mitochondria, and signs of mitophagy in SMS patient-derived cells. **A** Electronic micrographs illustrating the CTR (CTR1 and sibling) and SMS (RAI1-del1 and RAI1-S399P40fx) fibroblast morphology (entire cell). Representative images of 15 cells from three biological replicates are shown. Scale bar: 5 µm, magnifications: 2 µm and 500 nm. Nucleus (N). **B**, **C** Quantification of the number of EVs per cell (top panel) and area (bottom panel) occupied by EVs (%) in SMS cells compared with control cells. **D** Electron micrographs showing mitochondria (M), endoplasmic reticulum (ER), swollen mitochondria (Smt), ER expansion (*), and autophagic vesicles (arrows) in control and SMS cells. Representative images of 15 cells from three biological replicates are shown. Scale bar: 5 µm. **E**, **F** Quantification of swollen mitochondria (Smt, **E**) per cell and endoplasmic reticulum area (ER, **F**) relative to the nucleus in CTR and SMS cells (*n* = 15 cells from three biological replicates). **G** Quantification of the MitoTracker FACS analysis results revealed reduced mitophagy in CTR and SMS cells (*n* = 5–7 biological replicates). Graphs: mean ± SEM, one-way ANOVA + Newman–Keuls post hoc test, *p < 0.05, **p < 0.01, ***p < 0.001.

We subsequently performed an ultrastructural electron microscopy analysis of SMS and CTR cells. We found a significantly higher (2- to 3-fold) average number of cytoplasmic vacuoles with an ‘empty’ appearance (EVs) in RAI1-S399P40fx and RAI1-del1 cells than in CTR cells (Fig. 6A, B). Consistent with these findings, the percentage of cytoplasmic area occupied by EVs was 5% in SMS cells and 1–2% in CTR cells (Fig. 6C). The mitochondria were empty, fragmented, and had abnormal cristae (Fig. 6D). We found a significantly higher number of swollen mitochondria in RAI1-S399P40fx (approximately 9/cell) and RAI1-del1 cells (approximately 8/cell) than in control cells (approximately 1-2/cell) (Fig. 6E). The ER was often enlarged, with median ratios of the ER area to nucleus area of 5.9 in RAI1-S399P40fx cells and 7.4 in RAI1-del1 cells compared with 3–4 in CTR cells, which suggests potential ER stress (Fig. 6F). These ultrastructural analyses revealed that loss of *RAI1* causes accumulation of EVs in the cytosol and alters the mitochondrial morphology.

Defective mitochondria are eliminated through a special type of autophagy known as mitophagy [37]. For the assessment of mitophagy in living cells, we transduced the cells with a lentiviral vector expressing the pH-sensitive protein mt-Keima [38]. Fusion of damaged mitochondria with the lysosome results in transition from a basic physiological pH (pH 8) to an acidic pH (pH 4), which leads to a change in the excitation/emission wavelength of the reporter. Consistent with defects in autophagic flux (Fig. 5), we found that the level of mitophagy was significantly lower in the two SMS cell lines than in CTR cells (Fig. 6G). These observations suggest that SMS cells are unable to eliminate depolarized mitochondria, possibly due to defective autophagy.

### SMS cells show increased ROS production and cell death

**Fig. 7:**
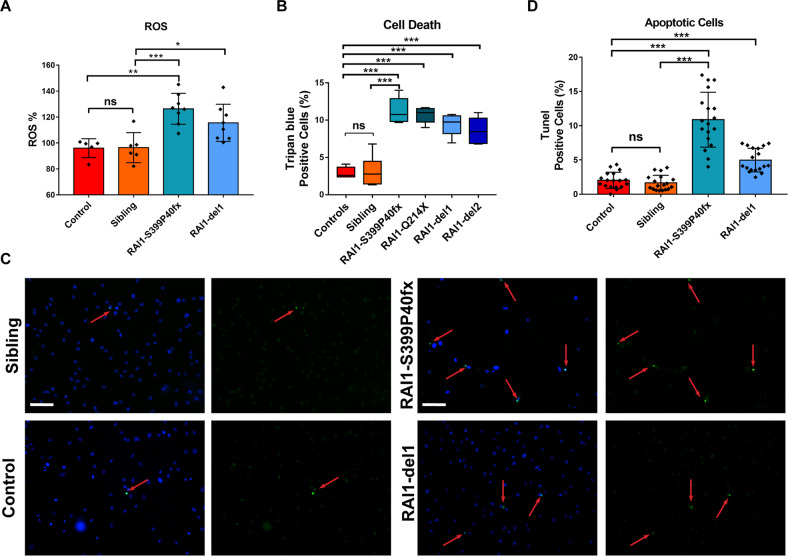
Loss of RAI1 results in increased ROS production and cell death. **A** Quantification of reactive oxygen species (ROS) measured through an analysis of 2,7-dichlorodihydrofluorescein diacetate (DCFDA) fluorescence in CTR (CTR1 and sibling) and SMS (RAI1-S399P40fx and RAI1-del1) cells. The data are expressed as percentages of the control values after normalization by the total protein content (*n* = 5 biological replicates). **B** Trypan blue analysis of four CTR and four SMS cell lines 24 h after seeding (*n* = 5 biological replicates). **C**, **D** TUNEL assay of CTR (CTR1 and sibling) and SMS (RAI1-S399P40fx and RAI1-del1) cells. Representative images of 100 cells from three biological replicates are shown. Graphs: mean ± SEM, unpaired t test, *p < 0.05, **p < 0.01, ***p < 0.001.

The accumulation of LDs, defective autophagy, and damaged mitochondria are often associated with an overproduction of ROS [39, 40]. Using the fluorescent probe 2´,7´-dichlorodihydrofluorescein diacetate (DCFDA), we found that the ROS levels were significantly increased by 2- and 1.6-fold in RAI1-S399P40fx and RAI1-del1 fibroblasts, respectively, compared with control cells (Fig. 7A). ROS accumulation may cause cell death [41]. Through Trypan blue staining, we found that the number of dead cells was approximately 12% and 6% in the RAI1-S399P40fx and RAI1-Q214X cells and 9% and 7% in the RAI1-del1 and RAI1-del2 cells, respectively, whereas a value of 3% was found for both the sibling and CTR cells (Fig. 7B). A TUNEL assay revealed that the percentage of apoptotic cells was increased by 10.5% and 4% in RAI1-S399P40fx and RAI1-del fibroblasts, respectively (Fig. 7C, D). These findings indicate that *RAI1* loss of function results in excessive ROS production and increased apoptosis.

### N-acetylcysteine modifies the SMS cell phenotype

**Fig. 8:**
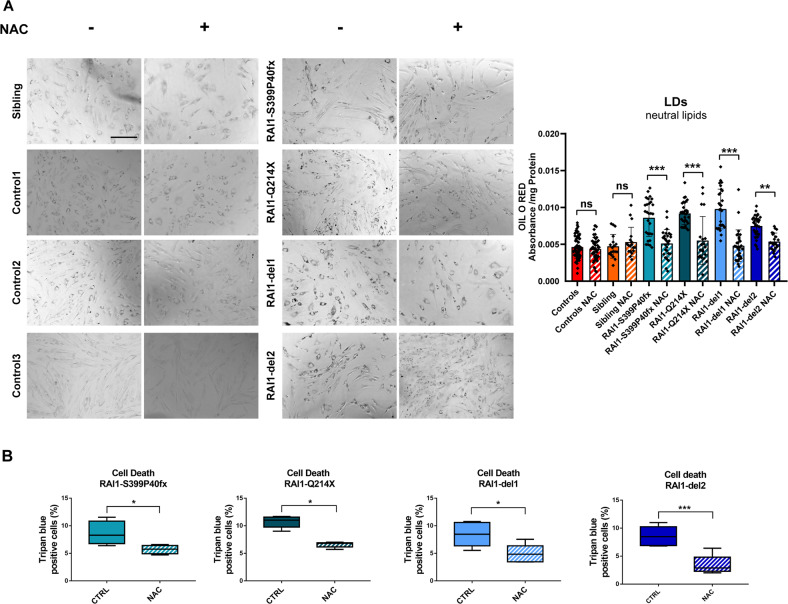
N-acetylcysteine attenuates the phenotype of SMS cells. **A** Left panel: Representative fluorescence microscopy images of lipid droplets (LDs) colored with Oil Red O in four SMS and four CTR cells (CTR1/2/3 and sibling) treated with N-acetylcysteine (NAC, 10 μM, 24 h) (*n* = 5 biological replicates). Right panel: Quantification of the optical density of 10 independent wells from five biological replicates. **B** Trypan blue analysis of cell death in four SMS and four CTR cell lines (CTR1/2/3 and sibling) treated with NAC (10 μM, 24 h) (*n* = 5 biological replicates). Graphs: mean ± SEM, (**B**) one-way ANOVA + Newman–Keuls post hoc test, (**C**) unpaired t test, *p < 0.05, **p < 0.01, ***p < 0.001, ns not significant.

Based on the above-described results, we tested the efficacy of N-acetylcysteine (NAC), which has a strong antioxidant capacity and regulates lipid metabolism [42]. Through Oil Red O staining, we found that NAC significantly reduced LD accumulation in all SMS cell lines tested in this study (Fig. 8A). Although NAC did not modify the accumulation of p62 and LC3 in the SMS cells (Supplementary Fig. 3), NAC significantly reduced the degree of SMS cell death (Fig. 8B). Collectively, these observations show that NAC modifies key aspects of SMS cell pathology, leading to increased cell viability, and thus highlight the potential beneficial effects of NAC on this incurable disorder.

## Discussion

Here, we provide the first evidence of pathological processes involving defects in lipid metabolism, mitochondrial morphology and autophagic flux from patient-derived cells. The reported findings were obtained from four independent cell lines and four control lines. Despite the tremendous genomic variability of human cells, we were nonetheless able to observe the following shared pathological features among the SMS cells: a deregulated expression of genes involved in lipid metabolism, lysosome function, and protein/lipid trafficking, accumulation of LDs, impaired autophagy/mitophagy flux, and increased cell death associated with ROS production. Moreover, based on our comparison of CNVs with *RAI1* point mutations, we propose that the phenotype described here is mainly due to *RAI1* haploinsufficiency.

Limited knowledge of the function(s) of the dosage-sensitive gene *RAI1* is mainly due to the lack of suitable models of SMS that can reproduce the main features of the disease [43]. Our approach was to use skin fibroblast lines obtained directly from patients carrying either *RAI1* point mutations or SMS CNVs and control lines from a healthy sibling and three unrelated donors. Our biopsies were obtained using the same procedure and were collected from the same point in the arm, and the experiments were all performed at well-defined passages. This design allowed us to reduce the variability that would be observed using cells of unknown origin, different passages and collected using different processes. By using standardized procedures and culturing protocols, we minimized the clone-to-clone variability intrinsic to human primary cells.

We found reduced RAI1 transcript and protein levels in the SMS cells carrying the CNV and the SMS cells with *RAI1* point mutations, as previously described [15]. It is possible that the decreases in the *RAI1* transcript levels found in the SMS point mutation cell lines is due to nonsense-mediated decay [44]. Another important aspect that could be relevant to disease is that premature stop codons may lead to the generation of a truncated protein with aberrant subcellular localization and function. We show that RAI1 is present in both the nucleus and the cytoplasm in CTR and SMS cells.

Our gene expression analysis revealed alterations in the expression levels of components of the sterol biosynthetic pathway and lysosomal enzymes. Mutations in several genes that were found to be dysregulated in SMS cells cause severe genetic developmental disorders. SQLE is a flavin adenine dinucleotide (FAD)-dependent enzyme that catalyzes the rate-limiting step in cholesterol conversion, and DHCR7 is a nicotinamide adenine dinucleotide phosphate (NADPH)-dependent enzyme that catalyzes the last step of the Kandutsch-Russell cholesterol biosynthetic pathway [22, 23, 45]. *SQLE* has been linked to Rett syndrome pathogenesis [46], and mutations in *DHCR7* result in a developmental disability known as Smith-Lemli-Lopiz Syndrome (SLOS, MIM 270400) [47], which resembles SMS in some aspects. N-acyl sphingosine amidohydrolase 1 (*ASAH1*) encodes the lysosomal acid ceramidase that hydrolyses the bioactive sphingolipid Cer into sphingosine and free fatty acids under acidic pH conditions, and loss-of-function mutations in this gene cause two different disorders, Farber disease (FD) and a rare form of spinal muscular atrophy combined with progressive myoclonic epilepsy (SMA-PME) [48, 49]. Hexosaminidase subunits alpha and beta (*HEXA* and *HEXB*) form a glycosyl hydrolase in lysosomes and catalyze the degradation of the ganglioside GM2, and loss-of-function mutations in these genes lead to Tay–Sachs disease (GM2-gangliosidosis type I) and Sandhoff disease (GM2-gangliosidosis type II) [50, 51]. These disorders are characterized by the accumulation of GM2 ganglioside in lysosomes mainly in neurons and are characterized by neurodevelopmental alterations associated with neuronal inflammation and apoptosis [52]. RAI1 haploinsufficiency has previously been associated with altered gene expression. In particular, as a transcription factor, its haploinsufficiency induces a general downregulation of gene expression [6, 15]. Moreover, RAI1 transcriptional activity is influenced by neuronal activity [53]. We observed a significant number of upregulated genes, which are the result of pathological processes occurring in these cells. In fact, the overexpression of genes whose function is conducted in lysosomes reflects the accumulation of dysfunctional lysosomes, as demonstrated by the LysoTracker experiment. Nonetheless, the observation that the genes dysregulated in SMS cells are linked to developmental diseases suggests that an altered lipid metabolism is a common pathogenetic pathway in neurodevelopmental diseases such as SMS.

To shed light on the pathogenic pathways underlying the phenotype of SMS cells, we combined our transcriptome analysis with lipidomic analysis. SMS cells were characterized by high levels of TGs and an alteration of the sphingolipid pathway. In particular, the levels of Cer, sphingosine, and GM2 were decreased, and the levels of GD3 were increased. We noticed that the decrease in the GM2 level is consistent with the both the overexpression of HEXA/HEXB identified by transcriptomic analysis and the aberrant accumulation of lysosomes inside the cytoplasm. The accumulation of TGs is consistent with the fact that *RAI1* haploinsufficiency is strongly associated with a tendency toward obesity in SMS patients. Importantly, SMS patients show higher levels of total cholesterol, high-density lipoprotein (HDL), low-density lipoprotein (LDL), and TGs [54]. The most widely accepted hypothesis is that RAI1 haploinsufficiency causes compulsive hyperphagic behavior, which is the main cause of obesity in patients, and this aspect has been replicated in animal models of disease [2, 5, 6, 55, 56]. Our findings, however, reveal an alternative and possibly coexisting scenario: SMS cells are fat and exhibit lipid deregulation driven not only by nutrient availability but also by TG accumulation in LDs. Under normal conditions, cells use TGs as a source of energy when needed. Under pathological conditions, to avoid toxicity due to excessive accumulation, TGs are safely stored as neutral lipids in specialized organelles that can be eliminated through a type of autophagy, namely, lipophagy [57]. The number of LDs in SMS cells is substantially high and is not influenced by the addition of OA. This phenomenon may be the response through which the cells cope with excessive TG accumulation and suggests that LD accumulation is due to autophagic deregulation. This hypothesis is supported by the observed reduction in the phosphatidylinositol content in SMS cells, which suggests that the capacity of these cells to initiate the autophagy process is reduced. The phosphorylated form of phosphatidylinositol, namely, phosphatidylinositol-3-phosphate (PI3P), is a central lipidic player in membrane dynamics and trafficking regulation [58, 59]. PI(3)P-enriched membranes recruit autophagy-associated PI3P-binding proteins and directly participate in the activation of the downstream ATG machinery, including lipidated LC3 (LC3-II), which leads to nucleation of the autophagosomal membrane [60]. The aberrant accumulation of GD3 in SMS cells suggests that these cells activate an autophagy program in response to stress conditions associated with enhanced accumulation of lipids. In fibroblasts, GD3 plays a key role in the initiation phase of autophagy, autophagosome biogenesis, and autophagosome maturation into autolysosomes [61]. We observed an accumulation of LC3-II and p62 in SMS cell lines, which highlights that the impairment of autophagic flux is a crucial aspect of the pathophysiology of SMS cells.

Electron microscopy revealed significantly higher vacuolization inside SMS cells compared with control cells. These EVs are LDs and empty autophagosomes/lysosomes that cells have not eliminated despite successfully clearing their contents, possibly due to a block of autophagy. This defective autophagic flux then overwhelms the system, which leads to increased EV accumulation and thus chronically alters cell homeostasis. A gene that is important for autophagy and lysosome function is SMS chromosome region candidate gene 8 (*SMCR8*), which is deleted in CNV SMS patients. SMCR8 promotes the maturation of autophagosomes and ensures lysosome function [62]. Loss of function of this gene may contribute to autophagy defects in SMS. However, this gene is missing only in RAI1-del1 cells. Thus, the autophagy defects observed in the SMS cell lines used in this study are unlikely to be associated with the loss of *SMCR8*. Another interesting candidate modifier gene is sterol-regulatory element-binding factor 1 (*SREBF1*), which translates signals to the nucleus and acts as a lipid-activated transcription factor [54]. *SREBF1* is deleted in some CNV SMS patients and thus likely contributes to phenotype. However, this gene is present in all SMS cells included in this study. We also observed a significant increase in swollen mitochondria, enlarged ER and reduced mitophagy in SMS cells. The presence of dysfunctional mitochondria could be explained by the accumulation of GD3, which induces dissipation of the mitochondrial membrane potential and the swelling of isolated mitochondria and thus results in the release of cytochrome *c*, apoptosis-inducing factor, and caspase 9 activation [61, 63]. We speculate that the increased ROS levels could be linked to a failure of the elimination of impaired mitochondria. All these features can be correlated with either impairment of autophagic flux due to it being overwhelmed by the accumulation of LDs/EVs or impairment of lipid metabolism that damages the capacity of cells to eliminate supernumerary vesicles.

One medication currently administered for SMS patients is melatonin, which is given to alleviate sleep disorders and exerts antioxidant effects [64]. We found that the treatment of SMS cells with NAC attenuates two pathological features of SMS cells, namely, LD accumulation and cell death. Interestingly, the effect of NAC on cell death was found to be associated with a reduction in the accumulation of LDs rather than an overt effect on autophagy flux, which highlights the relevance of lipid dyshomeostasis and LD accumulation in SMS. NAC is a potent antioxidant that reduces or prevents oxidative stress by working as a reductant, scavenger and precursor in glutathione biosynthesis. NAC reduces LD accumulation in stem cells and in adipocytes derived from bone marrow stromal cells [65]. We propose that *RAI1* haploinsufficiency results in increased ROS production, accumulation of TGs in LDs, oxidative stress and apoptosis. Although whether NAC is effective in vivo remains to be established, our findings support the notion that NAC has therapeutic potential for SMS patients.

## Supplementary information

checklist

Merged figure file

certification of English editing

Suppl.Fig.1

Suppl.Fig.2

Suppl. Fig.3

Suppl.Table1

Suppl.Table 2

Suppl.Table 3

Suppl.Table 4

Legends of supplemental material

western blot uncutted

## Data Availability

GeneChip Human Transcriptome Array (HTA) 2.0 data were deposited into ArrayExpress under the accession number E-MTAB-10118.

## Materials availability

This study generated new unique SMS fibroblast lines.
