# Supplementary figures and images for: Retinoic acid-induced 1 gene haploinsufficiency alters lipid metabolism and causes autophagy defects in Smith-Magenis syndrome
Source: Cell Death Dis. 2022 Nov 21;13(11):981. doi: 10.1038/s41419-022-05410-7 (PMC9678881; doi:10.1038/s41419-022-05410-7)

**Figure 1**

**
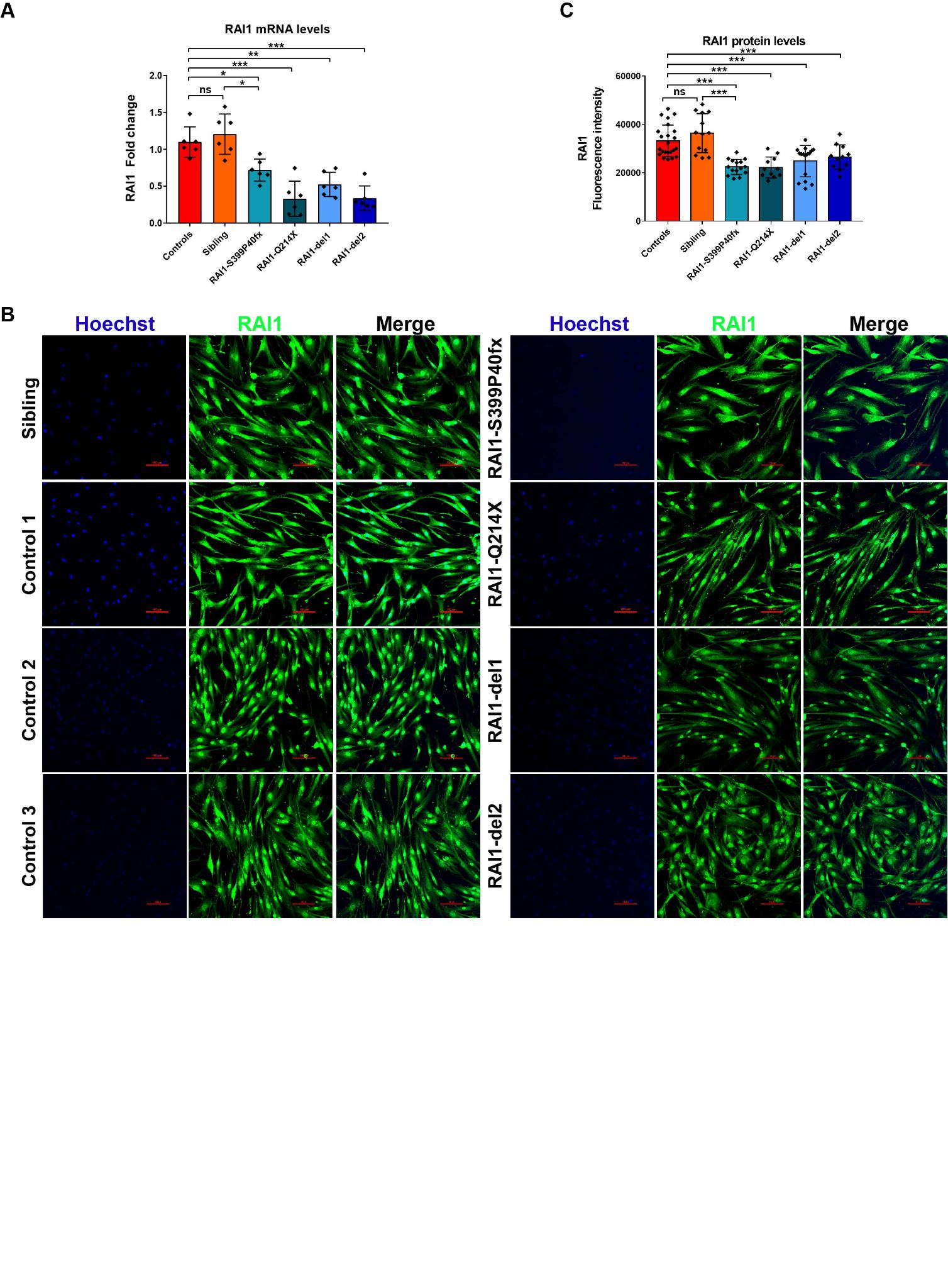
**

**Figure 2**

**
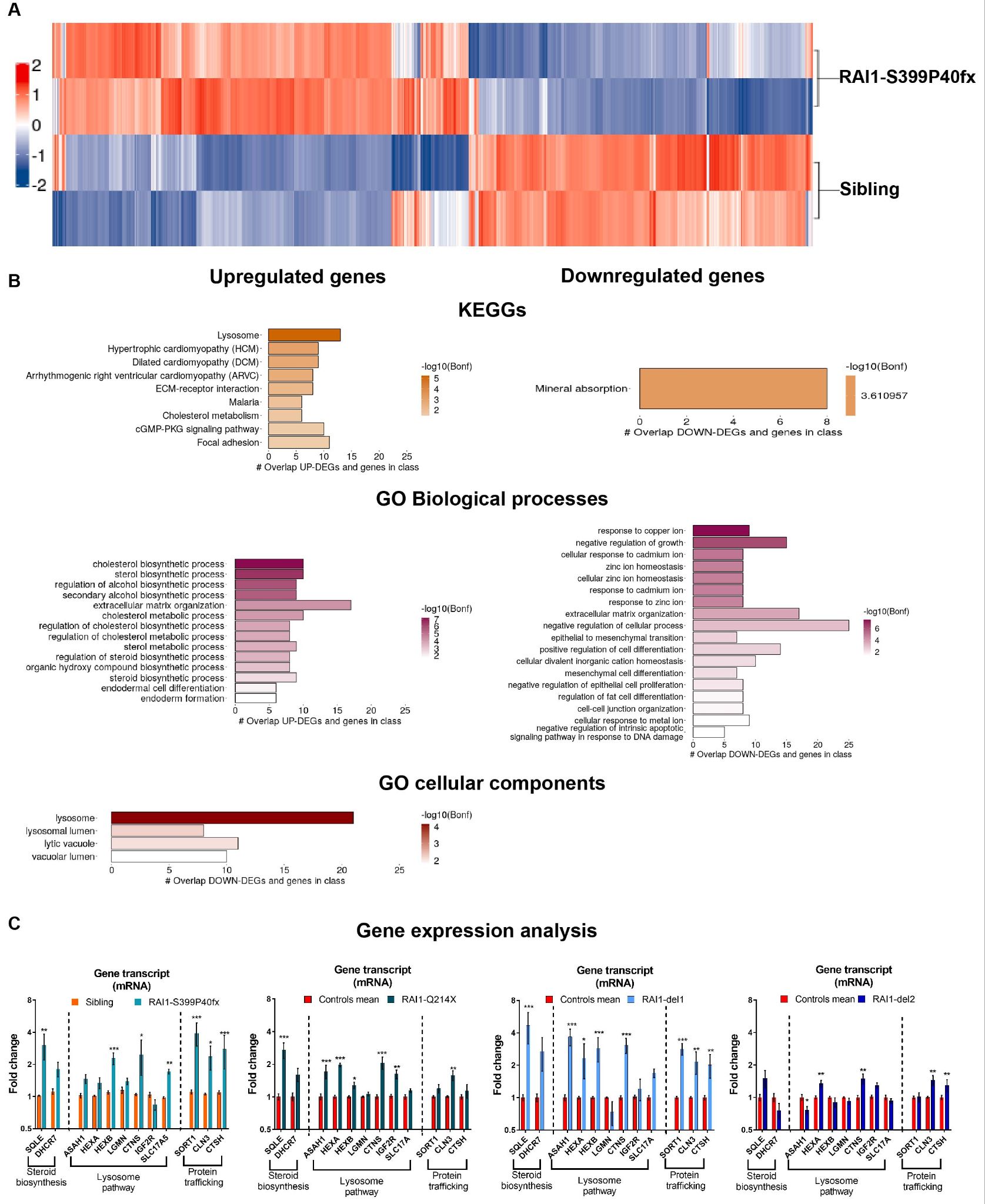
**

**Figure 3**

**
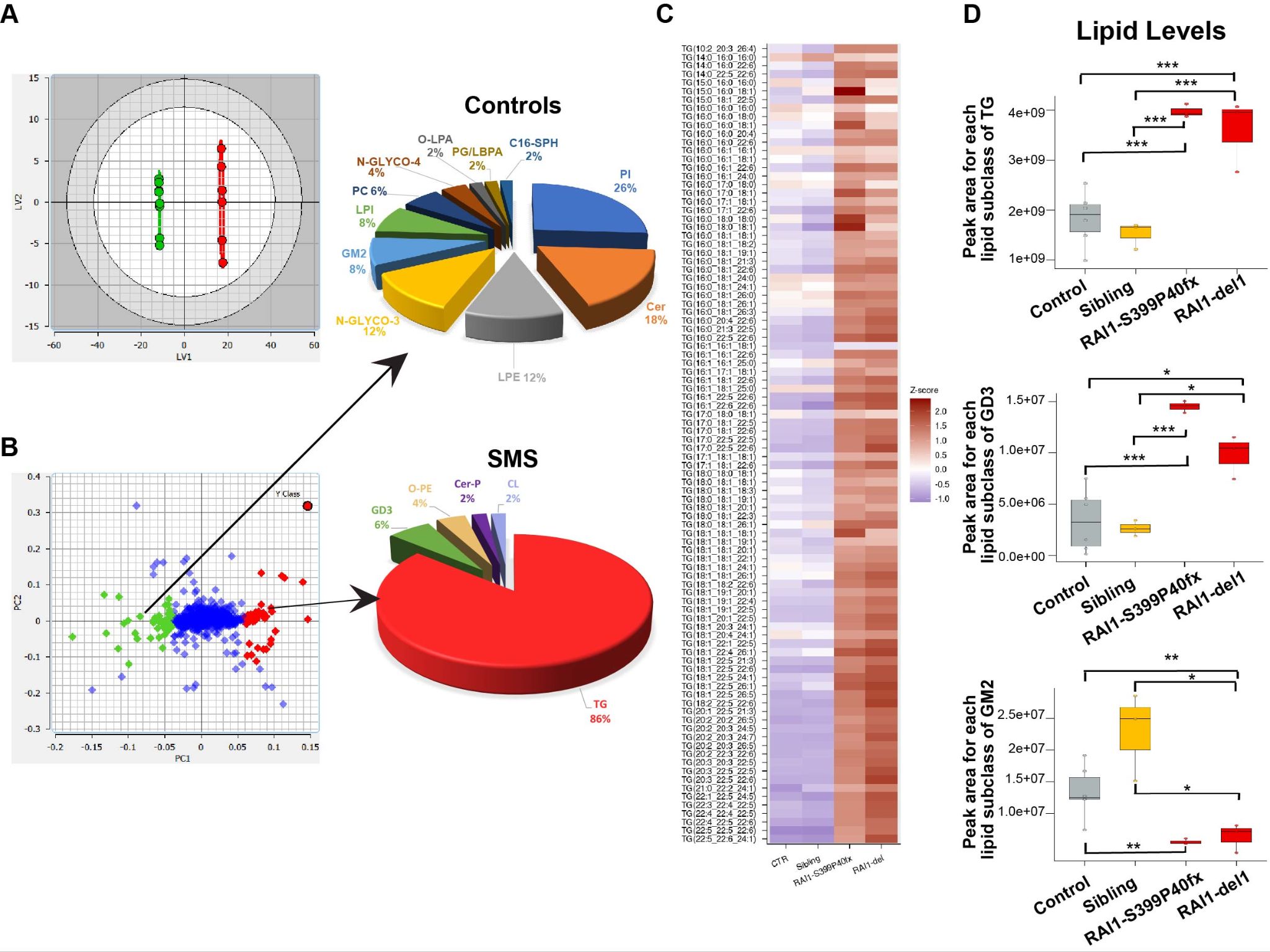
**

**Figure 4**

**
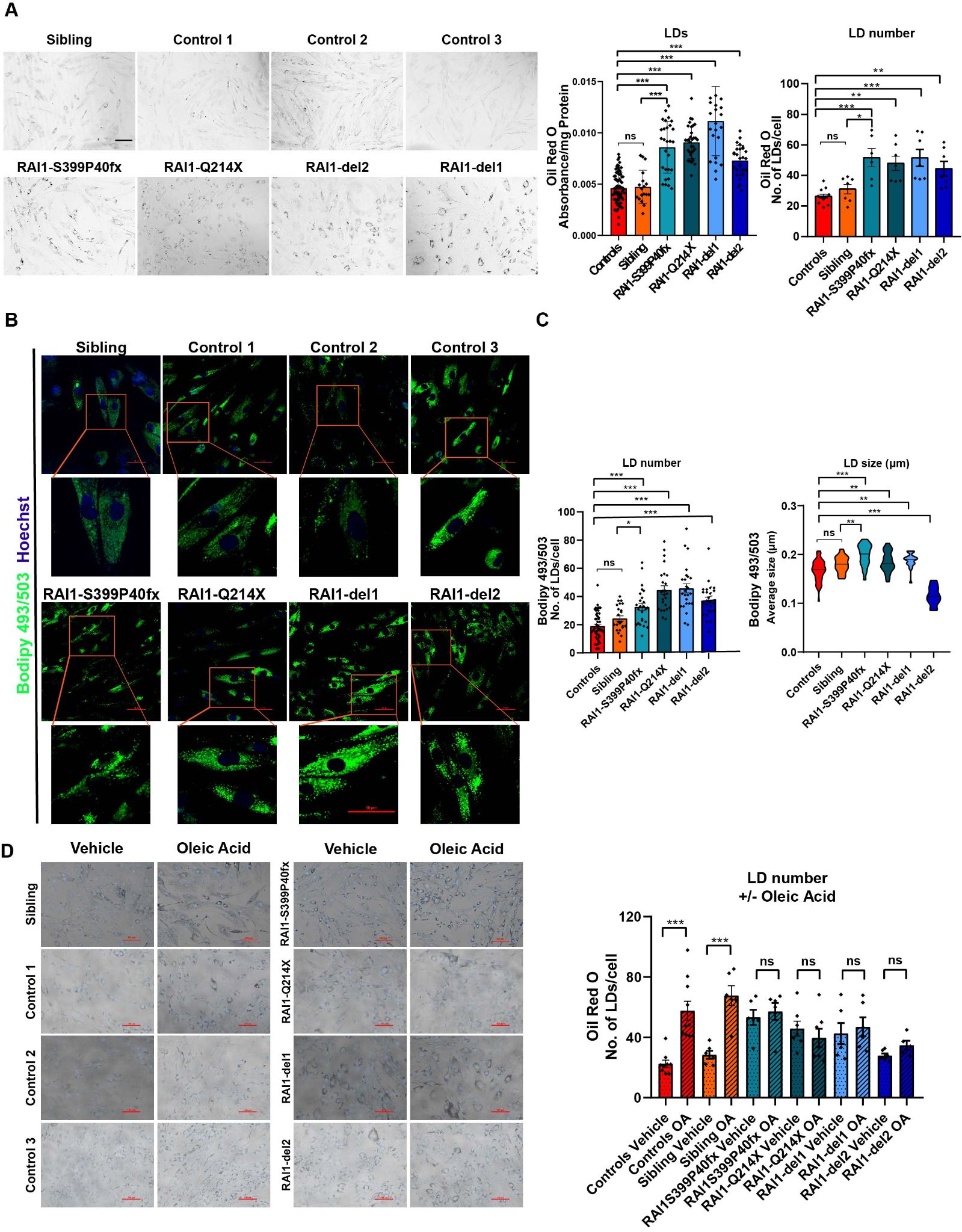
**

**Figure 5**

**
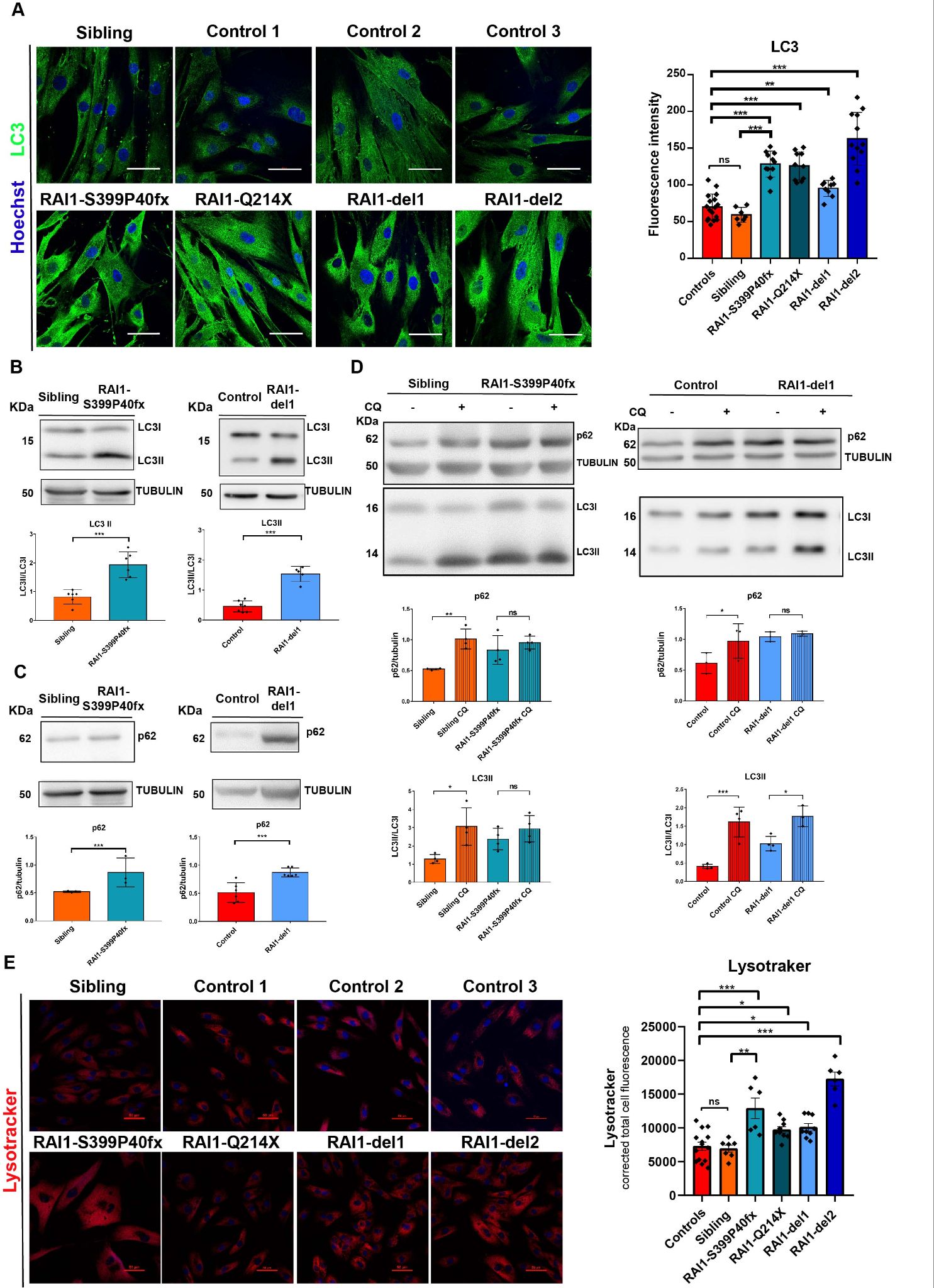
**

**Figure 6**

**
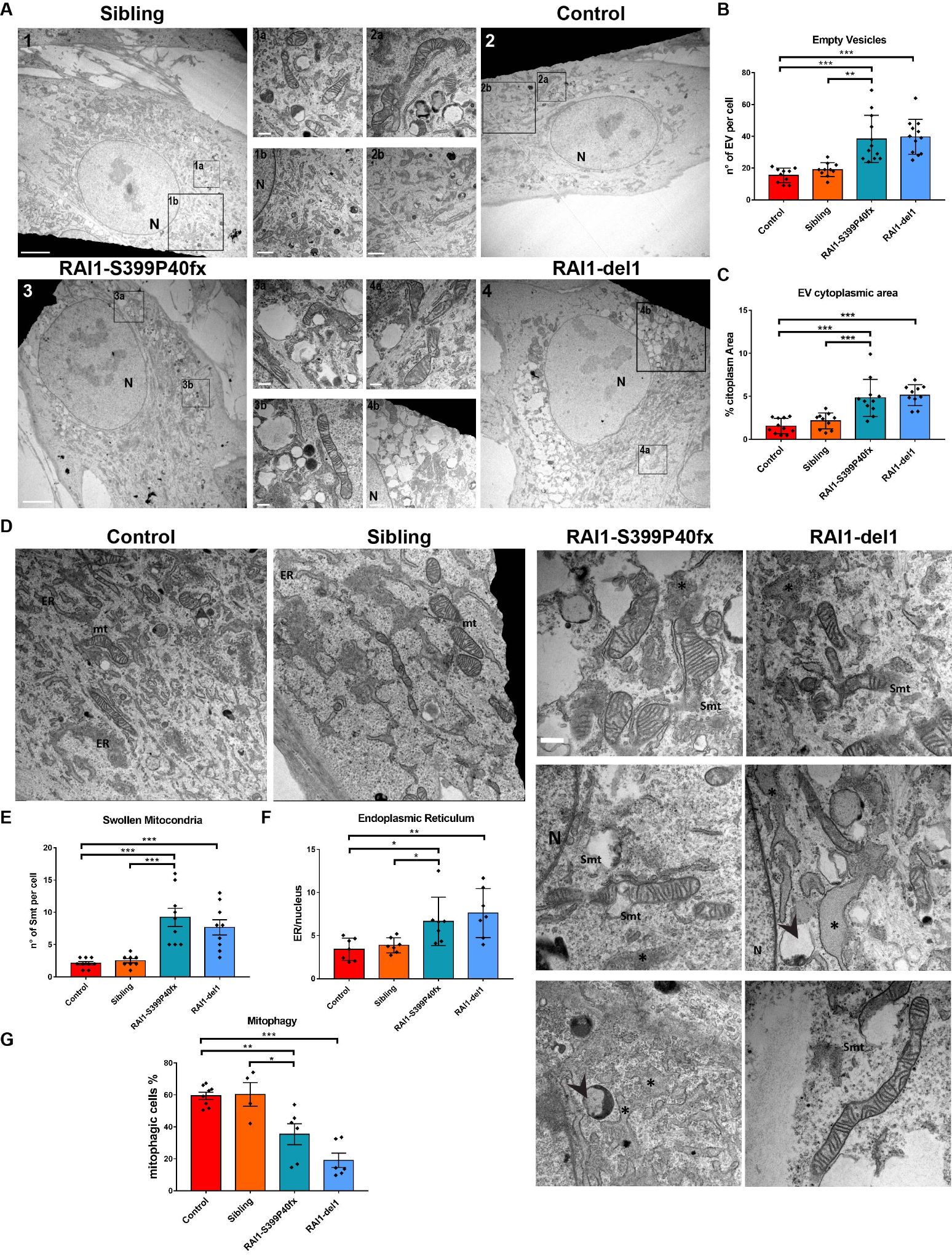
**

**Figure 7**

**
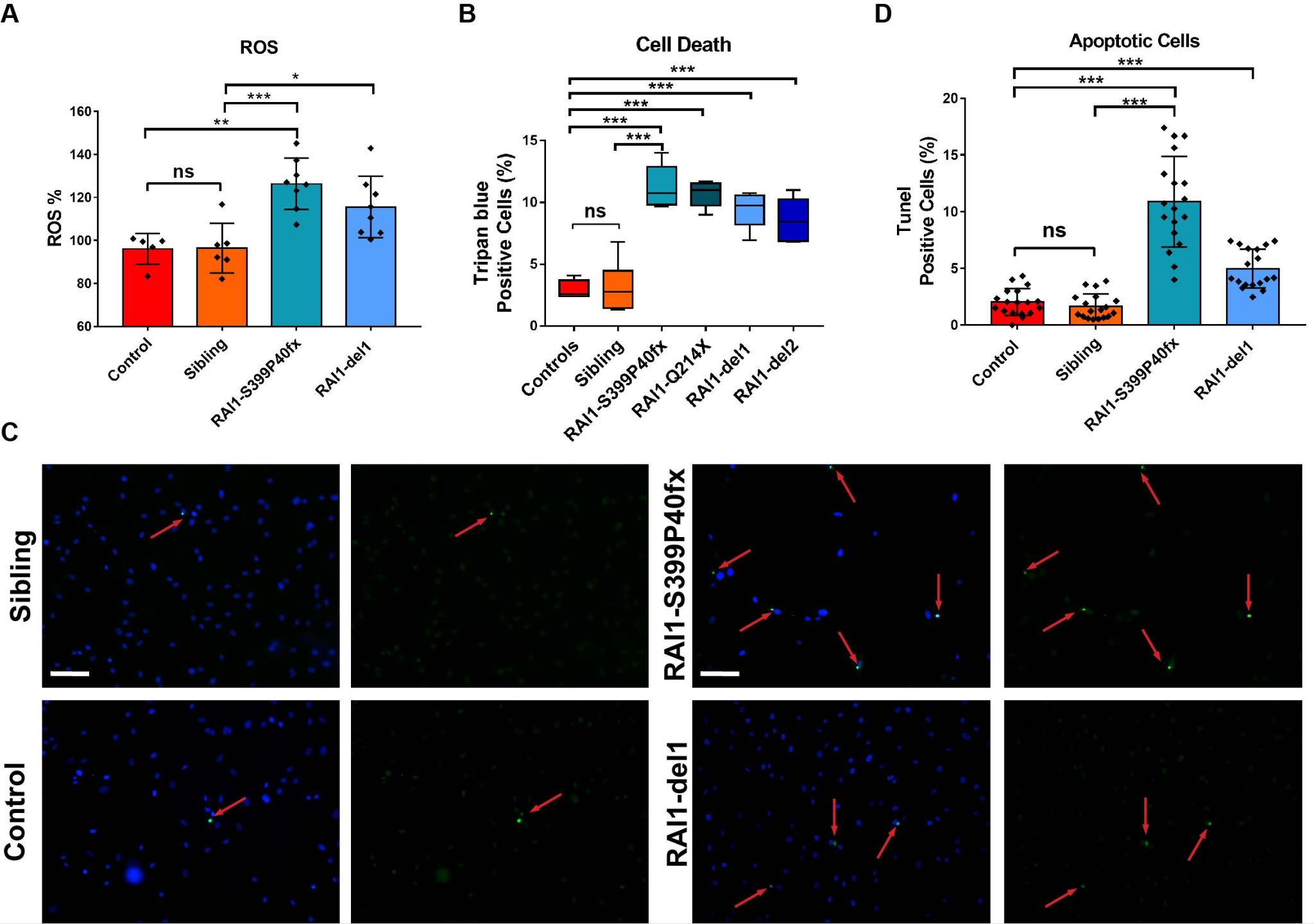
**

**Figure 8**

**
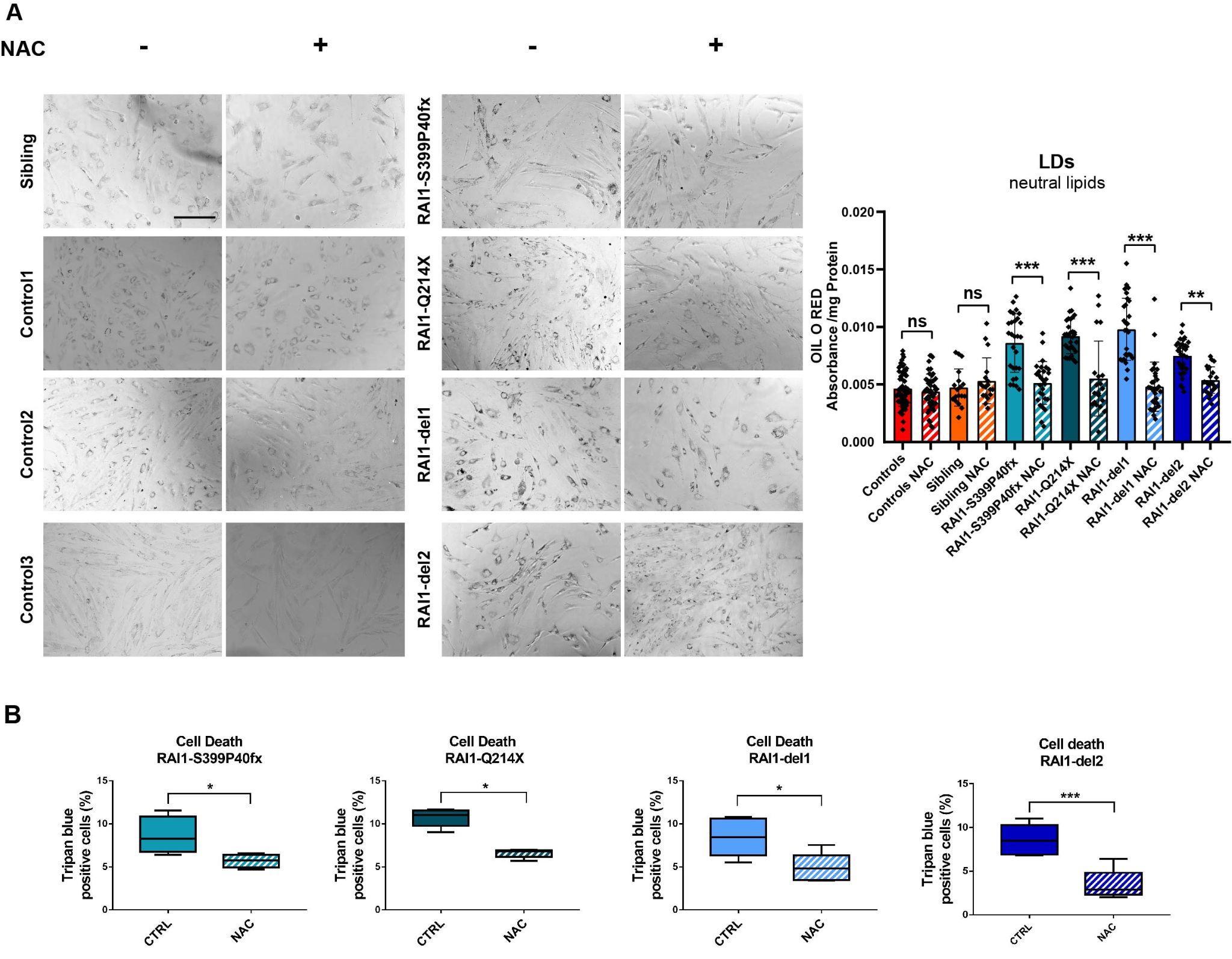
**

Supplement: Supplementary file 2 — Merged figure file [file 41419_2022_5410_MOESM2_ESM.docx]

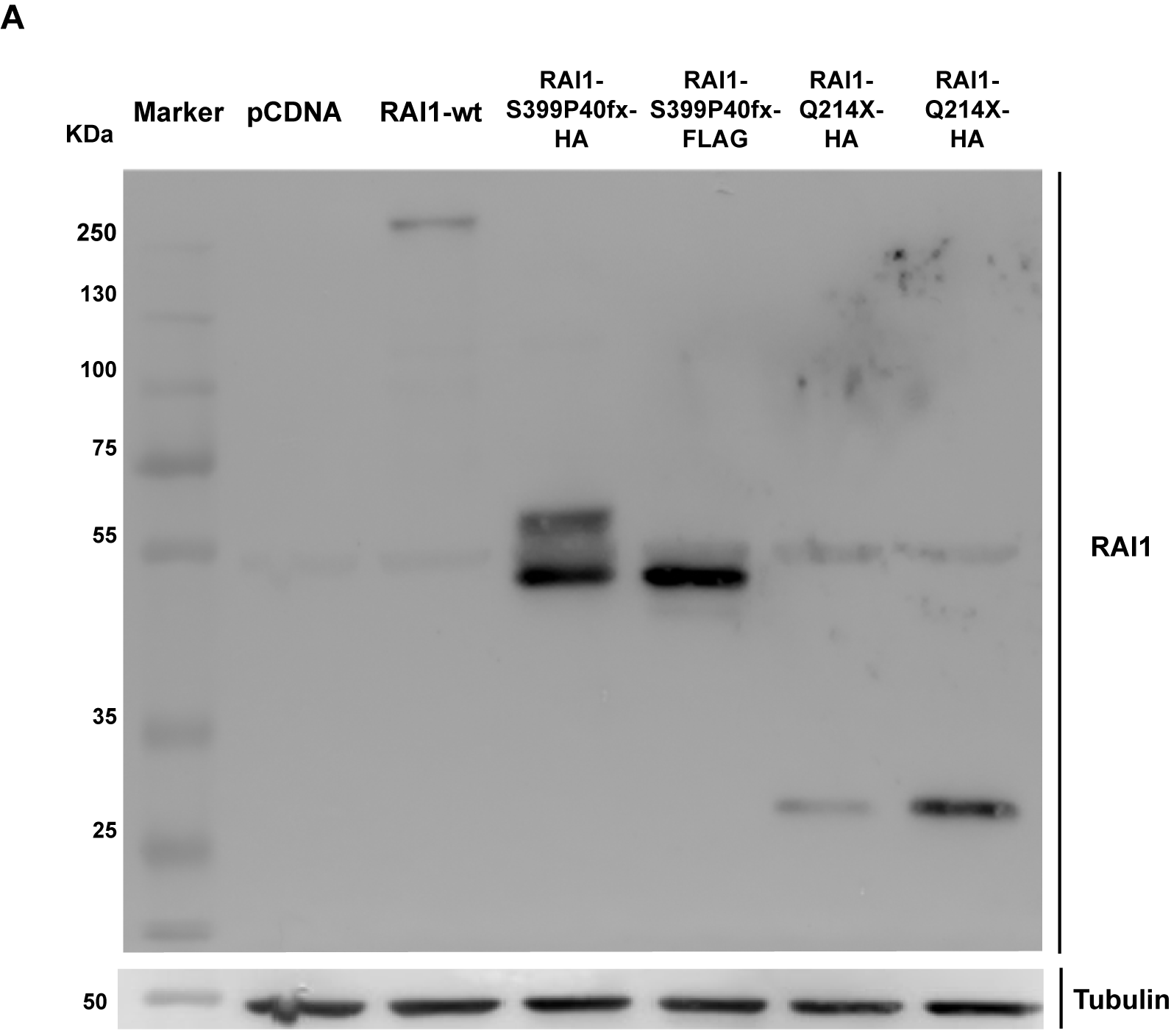

Supplement: Supplementary file 4 — Suppl.Fig.1 [file 41419_2022_5410_MOESM4_ESM.tif]

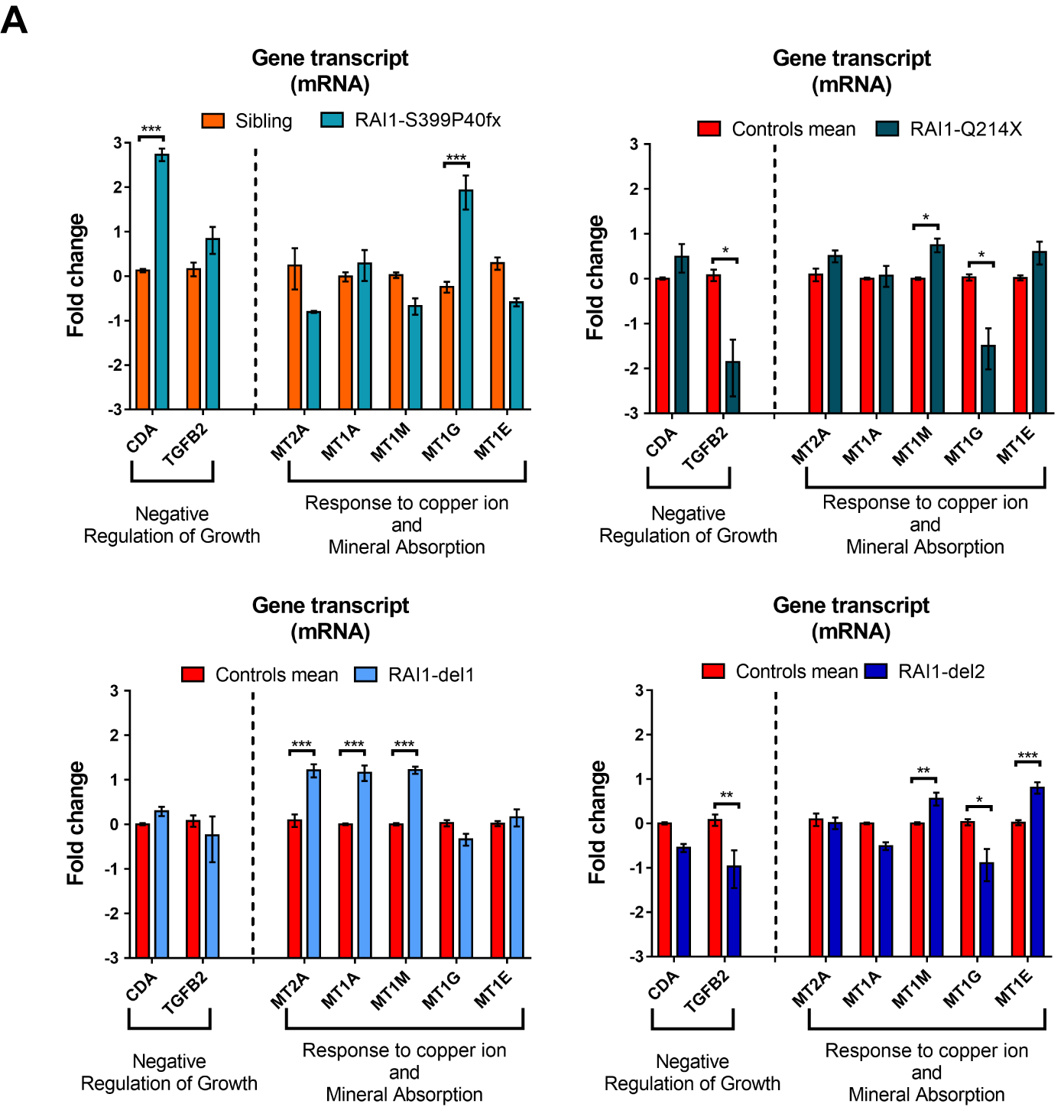

Supplement: Supplementary file 5 — Suppl.Fig.2 [file 41419_2022_5410_MOESM5_ESM.tif]

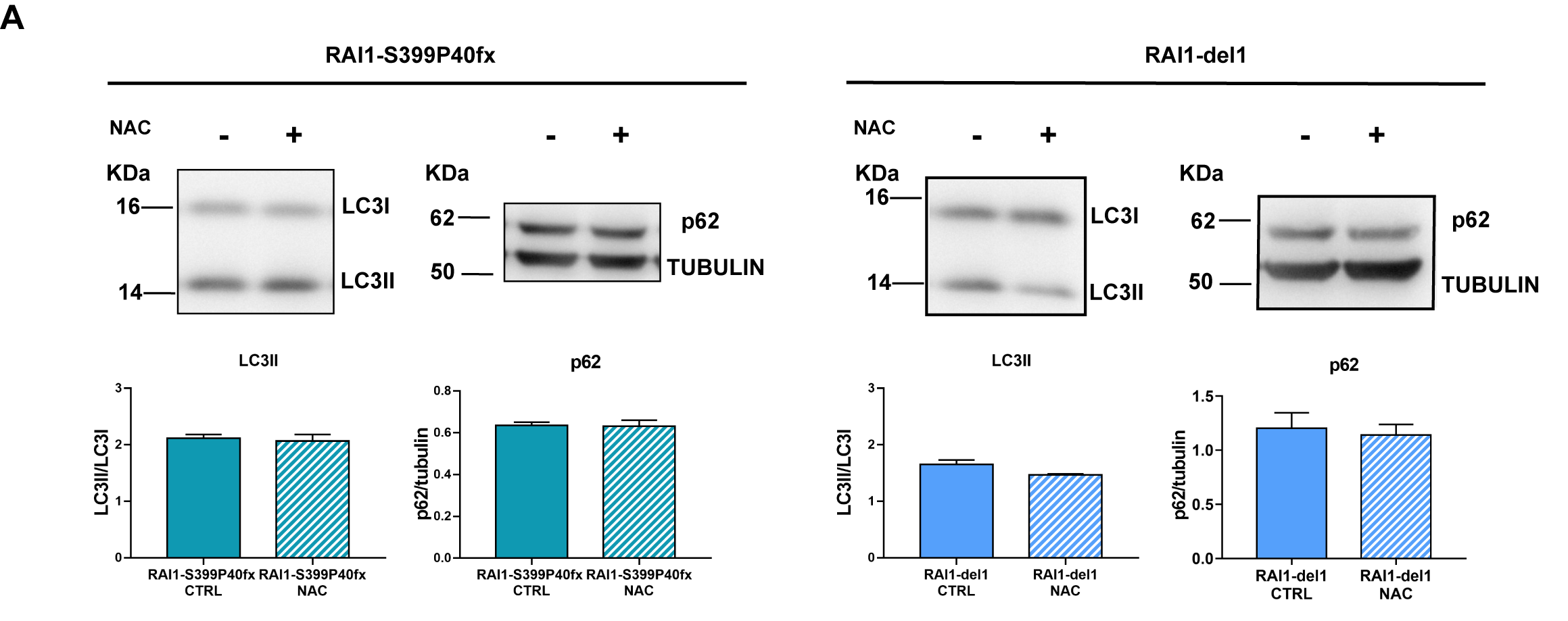

Supplement: Supplementary file 6 — Suppl. Fig.3 [file 41419_2022_5410_MOESM6_ESM.tif]
