# Supplementary material for: Retinoic acid-induced 1 gene haploinsufficiency alters lipid metabolism and causes autophagy defects in Smith-Magenis syndrome
Source: Cell Death Dis. 2022 Nov 21;13(11):981. doi: 10.1038/s41419-022-05410-7 (PMC9678881; doi:10.1038/s41419-022-05410-7)
Supplement: Supplementary file 11 — Legends of supplemental material [file 41419_2022_5410_MOESM11_ESM.docx]

**Retinoic acid-induced 1 gene haploinsufficiency alters lipid metabolism and causes autophagy defects in Smith-Magenis syndrome**

Elisa Maria Turco^1^, Angela Maria Giada Giovenale^1,2^, Laura Sireno^1,3^, Martina Mazzoni^1^, Alessandra Cammareri^1^, Caterina Marchioretti^3^, Laura Goracci^4^, Alessandra Di Veroli^4^, Daniel D’Andrea^5^, Elena Marchesan^6^, Barbara Torres^7^, Laura Bernardini^7^, Maria Chiara Magnifico^8^, Alessio Paone^8^, Serena Rinaldo^8^, Matteo Della Monica^9^, Stefano D’Arrigo^10^, Diana Postorivo^11^, Anna Maria Nardone^11^, Giuseppe Zampino^12,13^, Roberta Onesimo^12,13^, Chiara Leoni^13^, Federico Caicci^14^, Domenico Raimondo^15^, Elena Binda^16^, Laura Trobiani^17^, Antonella De Jaco^17,18^, Ada Maria Tata^17,18^, Daniela Ferrari^2^, Francesca Cutruzzolà^8^, Gianluigi Mazzoccoli^19^, Elena Ziviani^6^, Maria Pennuto^3*^, Angelo Luigi Vescovi^1,2*^, Jessica Rosati^1*^.

1. Cellular Reprogramming Unit, Fondazione IRCCS Casa Sollievo della Sofferenza, Viale dei Cappuccini, 71013 San Giovanni Rotondo (FG), Italy

2. Department of Biotechnology and Biosciences, University of Milano-Bicocca, P.zza della Scienza,2, 20126 Milan, Italy

3. Department of Biomedical Sciences, University of Padova, via Ugo Bassi 58/B, 35131 Padova, Italy; Veneto Institute of Molecular Medicine (VIMM), via Orus 2, 35129 Padova, Italy

4. Department of Chemistry, Biology, and Biotechnology, University of Perugia, Via Elce di Sotto 8, 06123 Perugia, Italy

5. Interdisciplinary Biomedical Research Center, School of Science and Technology, Nottingham Trent University, Clifton, NG11 8NS, UK.

6. Department of Biology, University of Padova, Via U. Bassi 58/b, 35121 Padova, Italy

7. Medical Genetics Unit, Fondazione IRCCS Casa Sollievo della Sofferenza, Viale dei Cappuccini, 71013 San Giovanni Rotondo, Italy

8. Department of Biochemical Sciences, “A.Rossi Fanelli”, University of Rome "La Sapienza", P.le Aldo Moro 5, 00185 Rome, Italy

9. UOC Genetica Medica e di Laboratorio, *AORN "A. Cardarelli"*, Via Antonio Cardarelli 9, 80131 Napoli, Italy

10. Department of Pediatric Neuroscience, Fondazione IRCCS Istituto Neurologico Carlo Besta, Via Giovanni Celoria, 11, 20133 Milano, Italy

11. Medical Genetics Laboratory, "Policlinico Tor Vergata" Hospital, Viale Oxford 81, 00133 Rome, Italy

12. Rare Diseases and Birth Defects Unit, Fondazione Policlinico Universitario Agostino Gemelli IRCCS, Largo Agostino Gemelli 8, 00168 Rome, Italy

13. Dipartimento di Scienze della Vita e Sanità Pubblica, Università Cattolica del S. Cuore, Largo Francesco Vito, 1, 00168 Rome Italy

14. Department of Biology, DiBio Imaging Facility, University of Padova, Via U. Bassi 58/b, 35121 Padova, Italy

15. Department of Molecular Medicine, University of Rome “La Sapienza”, Viale Regina Elena 324, 00161 Rome, Italy

16. Unit of Cancer and Stem Cells, Fondazione IRCCS Casa Sollievo della Sofferenza, Viale dei Cappuccini, 71013 San Giovanni Rotondo (FG), Italy

17. Department of Biology and Biotechnology “Charles Darwin”, University of Rome “La Sapienza”, P.le Aldo Moro 5, 00185 Rome, Italy

18. Research Center of Neurobiology "Daniel Bovet", University of Rome “La Sapienza”, P.le Aldo Moro 5, 00185 Rome, Italy

19. Department of Medical Sciences, Division of Internal Medicine and Chronobiology Laboratory, Fondazione IRCCS Casa Sollievo della Sofferenza, Viale dei Cappuccini, 71013 San Giovanni Rotondo, Italy

* Corresponding authors: maria.pennuto@unipd.it, vescovia@gmail.com, j.rosati@css-mendel.it

**Suppl.Fig.1** **Anti-RAI1 antibody recognizes wt and mutant RAI1 overexpressed proteins.**

1. HEK293T were transfected with vectors expressing either wild type RAI1, RAI1-S399P40fx and RAI1-Q214X, and the expression of RAI1 was detected in total lysates.

**Suppl. Fig.2. RAI1 haploinsufficiency alters the expression of genes involved in negative regulation of growth and in mineral adsorption**

A) Quantitative real-time PCR of the indicated genes in SMS and control cells (n = 5/genotype).

Graphs: mean ± SEM, one-Way ANOVA + Newman-Keuls post-hoc test, *p < 0.05, ** P < 0.01, ***p < 0.001.

**Suppl. Fig. 3. Influence of NAC on authopagy flux in SMS cells**

A) NAC did not influence autophagy flux.

**Suppl.Table 1 Whole-genome microarray analysis identifies the differentially expressed genes (DEGs)**

**Suppl. Table 2. Composition of the total lipid fingerprint and relative to the two groups Controls and SMS cells.**

**Suppl. Table 3. Details and individual peak area measurements for all the identified lipids in each sample.**

**Suppl. Table 4. Composition of 50 most enriched lipids among the SMS cells and the 50 most enriched lipids among the control cells, classified in category, main class and subclass.**
