# Supplementary material for: Retinoic acid-induced 1 gene haploinsufficiency alters lipid metabolism and causes autophagy defects in Smith-Magenis syndrome
Source: Cell Death Dis. 2022 Nov 21;13(11):981. doi: 10.1038/s41419-022-05410-7 (PMC9678881; doi:10.1038/s41419-022-05410-7)

Figure 4 C
Sibling
RAI1-S399P40fx
LC3

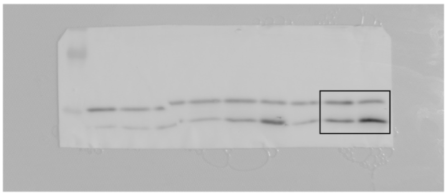

Figure 4 C
Control RAI1-del1
LC3

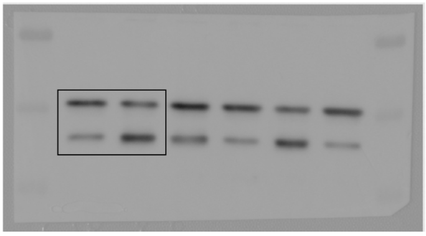

Figure 4 C
Sibling
RAI1-S399P40fx
Tubulin

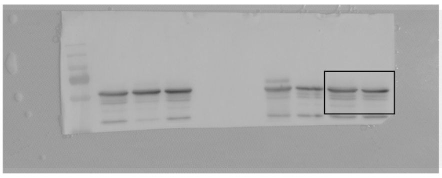

Figure 4 C
Control RAI1-del1
Tubulin

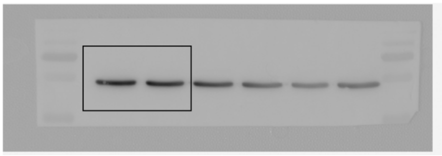

Figure 4 D
Sibling
RAI1-S399P40fx
p62

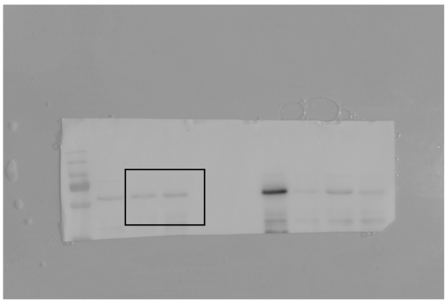

Figure 4 D
Control
RAI1-del1
p62 and
tubulin

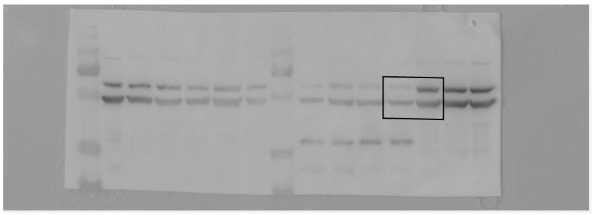

Figure 4
Sibling
RAI1-S399P40fx
tubulin

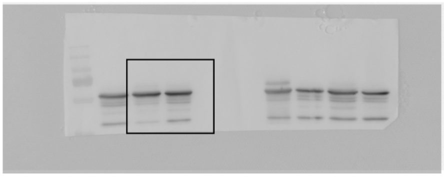

Figure 4 E
Sibling
RAI1-S399P40fx
LC3 +/- CQ

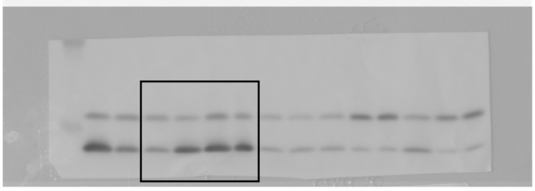

Figure 4 E
Control
RAI1-del1
LC3 +/- CQ

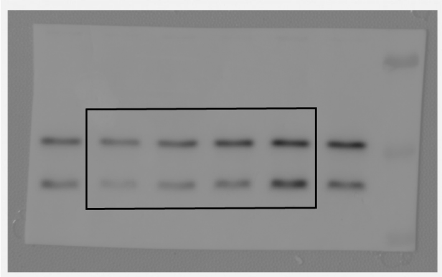

Figure 4 E
Sibling
RAI1-S399P40fx
p62 and tubulin
+/- CQ

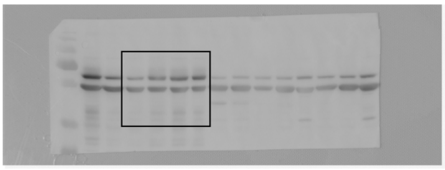

Figure 4 E
Control
RAI1-del1
p62 e tubulin
+/- CQ

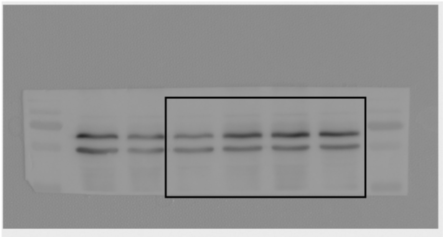

Supplementary
Figure 3 A
Sibling
RAI1-S399P40fx
p62 and tubulin
NAC

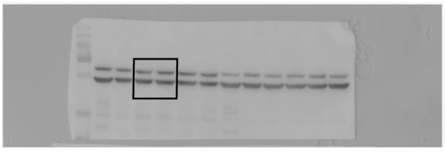

Supplementary
Figure 3 A
Control
RAI1-del1
p62 and tubulin
NAC

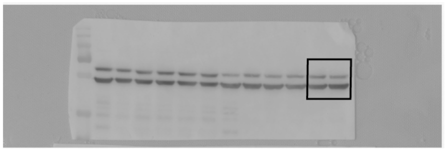

Supplementary
Figure 3 A
Sibling
RAI1-S399P40fx
LC3
NAC

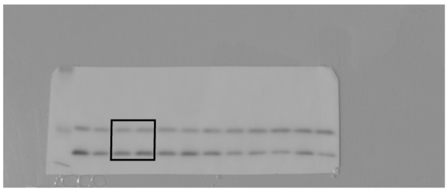

Supplementary
Figure 3 A
Control
RAI1-del1 LC3
NAC

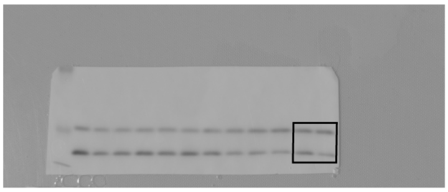

Supplement: Supplementary file 12 — western blot uncutted [file 41419_2022_5410_MOESM12_ESM.pdf]
